# Marine Actinobacteria a New Source of Antibacterial Metabolites to Treat Acne Vulgaris Disease—A Systematic Literature Review

**DOI:** 10.3390/antibiotics11070965

**Published:** 2022-07-18

**Authors:** Maria Clara De La Hoz-Romo, Luis Díaz, Luisa Villamil

**Affiliations:** 1 Doctoral Program of Biosciences, School of Engineering, Universidad de La Sabana, Chía 140013, Colombia; mariaderom@unisabana.edu.co (M.C.D.L.H.-R.); luis.diaz1@unisabana.edu.co (L.D.); 2 Bioprospecting Research Group, School of Engineering, Universidad de La Sabana, Chía 140013, Colombia

**Keywords:** biotechnology, marine actinobacteria, antibacterial activity, anti-biofilm activity, quorum-quenching activity, natural compounds, extracts, biosynthetic gene clusters (BGCs), acne vulgaris, *Cutibacterium acnes*, *Staphylococcus aureus*, *Staphylococcus epidermidis*

## Abstract

Acne vulgaris is a multifactorial disease that remains under-explored; up to date it is known that the bacterium *Cutibacterium acnes* is involved in the disease occurrence, also associated with a microbial dysbiosis. Antibiotics have become a mainstay treatment generating the emergence of antibiotic-resistant bacteria. In addition, there are some reported side effects of alternative treatments, which indicate the need to investigate a different therapeutic approach. Natural products continue to be an excellent option, especially those extracted from actinobacteria, which represent a prominent source of metabolites with a wide range of biological activities, particularly the marine actinobacteria, which have been less studied than their terrestrial counterparts. Therefore, this systematic review aimed to identify and evaluate the potential anti-infective activity of metabolites isolated from marine actinobacteria strains against bacteria related to the development of acne vulgaris disease. It was found that there is a variety of compounds with anti-infective activity against *Staphylococcus aureus* and *Staphylococcus epidermidis*, bacteria closely related to acne vulgaris development; nevertheless, there is no report of a compound with antibacterial activity or quorum-sensing inhibition toward *C. acnes*, which is a surprising result. Since two of the most widely used antibiotics for the treatment of acne targeting *C. acnes* were obtained from actinobacteria of the genus *Streptomyces*, this demonstrates a great opportunity to pursue further studies in this field, considering the potential of marine actinobacteria to produce new anti-infective compounds.

## 1. Introduction

Acne vulgaris is an inflammatory disease of the pilosebaceous unit that includes the hair follicle, hair shaft, and sebaceous gland. It is classified as a chronic condition due to the prolonged course and physical manifestations 1]. Furthermore, acne causes profound negative psychological and social effects on the quality of life of patients [1], affecting 85% of adolescents, and more than 10% of adults, and the Global Burden of Disease Project estimates that the prevalence of acne at 9.4%, placing it as the eighth most prevalent disease worldwide [2,3].

The pathophysiology of acne is related to the bacteria *Cutibacterium acnes*; this is one of the most abundant microorganisms found on human skin, accounting for up to 87% of the microorganisms in pilosebaceous units [4,5] along with *Staphylococcus epidermidis*, which are also major inhabitant Gram-positive bacteria of the skin microbiota. However, these bacteria adapt to changing skin microenvironments and can shift to being opportunistic pathogens, forming biofilms, and thus are involved in common skin dysbiosis, generating the loss of phylotype diversity of *C. acnes*, with the increase in pathogenic *Staphylococcus aureus* and commensal *S. epidermidis* [6].

Currently, all treatments available for the management of acne, topical or systemic, generate prominent side effects in patients such as psychiatric events, inflammatory bowel disease, hepatotoxicity, lupus-like syndrome, drug hypersensitivity syndrome, and so on [7]. Moreover, antibiotics have been a mainstay in the treatment of the disease; however, this has generated the emergence of antibiotic-resistant strains of *C. acnes*, which in turn exert selective pressure on other host bacteria such as *S. aureus* and *S. epidermidis*, allowing the emergence of antibiotic-resistant bacteria and contributing to bacterial drug resistance [8].

Thus, there is the need for new acne treatments to alleviate bacterial drug resistance, which has become a serious global threat to human health, food safety, animal production, and economic and agricultural development. Antibiotic resistance compromises the efficacy of prevention and treatment of infectious diseases, which are the number one cause of death in tropical countries, accounting for half of all fatalities. In addition, infectious disease mortality rates are also increasing in developed countries [9,10], and the lack of new antimicrobials against the spread of drug-resistant bacteria could generate 10 million deaths in the next 35 years. The prediction of losses of the order of 100 trillion USD is expected in 2050 if nothing is done to reverse the trend [11].

Since 2020, the World Health Organization, WHO (World Health Organization) has warned about the shortage of innovative antibiotics and their danger in treating drug-resistant infections [12,13]. As a result, novel approaches have emerged such as the use of bacteriophages, probiotics [14,15], and anti-biofilm agents/quorum-sensing inhibitors [16] a recommended alternative, since to date it is a mechanism against which bacteria have not shown resistance.

Historically, natural products isolated from a variety of sources such as terrestrial plants, animals, marine organisms, microorganisms, terrestrial vertebrates, and invertebrates have been a prolific source for numerous medical agents. In the early 20th century, approximately 80% of all medicines were obtained from plant sources. Nevertheless, since the discovery of penicillin from *Penicillium notatum* by Alexander Fleming in 1928, a significant shift from plants to microorganisms as a source of natural products has arisen [17].

Consequently, microorganism-derived compounds have been used based on a wide variety of biological activities. Among the bacteria, the actinobacteria phylum represents a noteworthy source of commercially important products and 70% of the known antibiotics are produced by actinobacteria, by the genus *Streptomyces* [18].

Most of these compounds were isolated from terrestrial sources [19]. Nevertheless, in the last 20 years, the re-discovery of previously characterized bioactive compounds and strain redundancy has decreased the interest in these soil-dwelling bacteria as a source of novel bioactive compounds. Therefore, Actinobacteria living in other niches, such as the marine environment (sea sediments, coral reefs, invertebrates, etc.), have gained value because of their chemo-diversity [18,19,20,21] influenced by their complex environment with extreme variations in pressure, salinity, light, and temperature [20].

It has been shown that marine actinobacteria exhibit more diverse and superior properties when compared to terrestrial actinobacteria in terms of antifouling, antibacterial, antibiofilm, anticoagulant, antiviral and antibacterial effects [19,22,23].

Since the marine actinobacteria have been less explored, here we did a systematic literature review of metabolites and extracts produced by marine actinobacteria with antimicrobial, anti-biofilm, and quorum-sensing inhibition activities (quorum quenching, QQ), as therapeutic alternatives treatment of acne vulgaris, some skin diseases, and infectious diseases.

Here we sort the reported metabolites regarding the type evaluated for their structure–activity relationship (SAR) and associated each family compound with some of their corresponding biosynthetic genes cluster. This systematic review aimed to assess the anti-infective potential of metabolites or compounds isolated from marine actinobacteria strains as an alternative treatment for acne vulgaris disease.

## 2. Results

### 2.1. General Information

**Figure 1:**
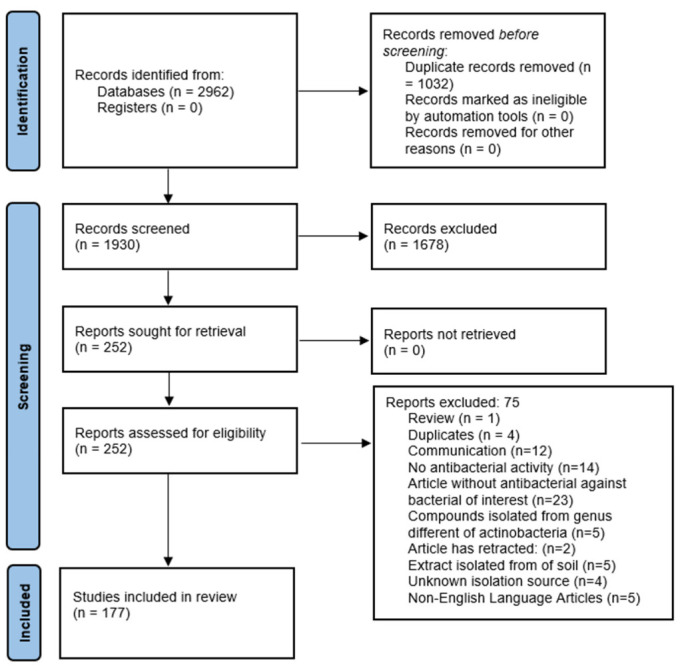
PRISMA flow diagram. Flowchart of systematic literature search according to PRISMA guidelines. Modified from [24]. The systematic review was done following the PRISMA guidelines, the complete checklist can be reviewed in Supplementary Table S1.

2962 articles were collected in this study and 1930 articles were identified after duplicate removal. Out of these, 1678 were excluded during the screening phase by title and abstract reading, and by applying the inclusion and exclusion criteria. Starting with this screening, 252 papers were selected for full-text reading, and they were assessed for eligibility. Finally, 177 papers were included for data extraction as shown in Figure 1.

### 2.2. Isolation Sources

**Figure 2:**
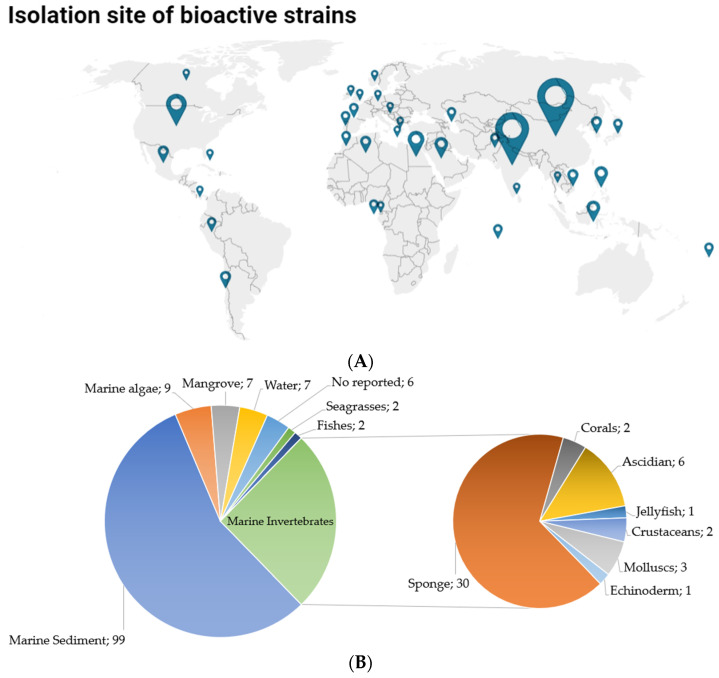
(**A**). World map showing the countries where marine actinobacteria with anti-infective activity were obtained. Max symbol size represents the number of reports. (**B**). Marine actinobacteria with anti-infective activity isolation sources.

The marine actinobacteria with anti-infective activity were collected worldwide, with a higher number of reports from China, (47), followed by India (39), and Egypt (10). In America, the United States was the most predominant country with 13 reported studies, followed by Mexico and Chile with 3 reports, Peru with 2, and Panama with 1. In the Caribbean, only one study was reported in the Bahamas and interestingly, some strains were isolated from oceans such as the Caspian Sea, the Baltic Sea, and the Cantabrian Sea, but not from the Bahamas maritime ecosystems (Figure 2A). Marine sediment was the most prevalent source with 99 of 177 studies reported, which was followed by isolation from sponges, with 30, and other marine invertebrates with 15 studies, such as sea squirts, corals, echinoderm-derived, mollusks, and jellyfish, as well as marine algae with 9 studies, water with 7, mangroves with 7, seagrasses with 2 and fishes with 2 (Figure 2B).

**Figure 3:**
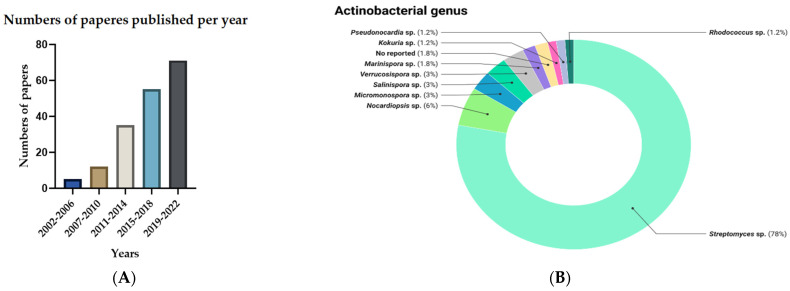
(**A**). Number of works related to marine actinobacteria per year organized by four-year period. (**B**) Actinobacterial genus reported in the studies of marine actinobacteria.

Likewise, when analyzing the number of articles published per year, a growth trend was evident; although the search of papers was not restricted by date, the oldest article was from 2002 and the most productive period was 2019 to 2022 (Figure 3A).

*Streptomyces* was the genus most reported with anti-infective activity in 78% of the published articles, followed by *Nocardiopsis*, *Micromonospora*, *Salinospora*, and *Verruscosispora* (Figure 3B). Likewise, some genera only were reported in 1% of papers such as *Actinomadura*, *Microbacterium*, *Micrococcus*, *Rothia kristinae*, *Brachybacterium*, *Serinicoccus* and *Solwaraspora* as presented in Figure 3B.

In addition to axenic culture, obtaining compounds from co-cultures has also been described. In total, 4 of 177 papers reported the use of co-cultures, where the most used genus was *Streptomyces*, followed by *Micromonospora* and *Actinokinespora* [25,26,27,28].

### 2.3. Organic Solvents Used to Obtain Anti-Infective Extracts

**Figure 4:**
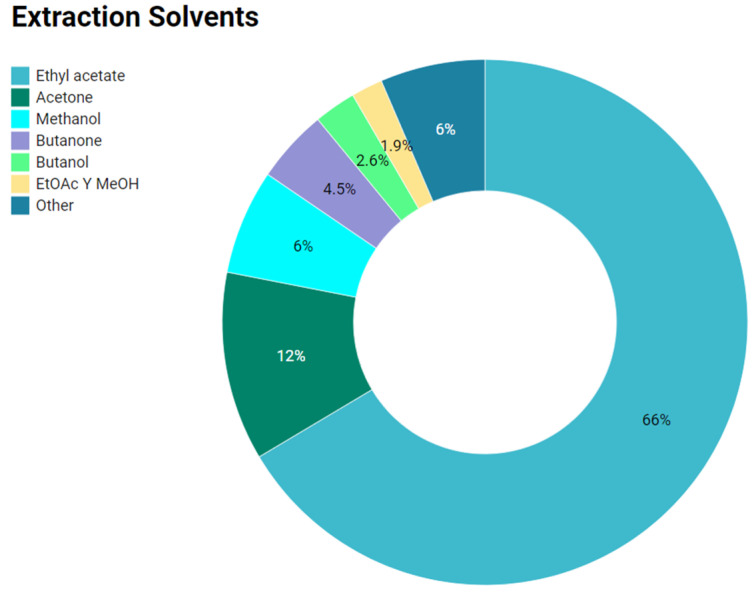
Organic solvents used to obtain actinobacterial extracts and compounds. EtOAc—MeOH: Ethyl acetate—Methanol.

Extracts and compounds with anti-infective activity have been isolated with different organic solvents; among them, ethyl acetate (EtOAc) is the most frequent. It was used in 66% of studies included in this review, followed by acetone, methanol (MeOH), butanone, butanol, and to a lesser extent dichloromethane, chloroform, ammonium sulfate, etc. Likewise, some solvents combinations have been reported. The most common are butanone–acetone and EtOAc–MeOH, among others. Figure 4 shows this distribution.

### 2.4. Anti-Infective Metabolites Derived from Marine Actinobacteria

It is necessary to indicate that here the anti-infective activity refers to a term that includes bacteriostatic, bactericidal, and quorum-quenching activity, which may interfere with virulence factors production, as well as biofilm formation. QQ is not involved in the pathogen elimination or reduction of planktonic cell growth, which may reduce drug resistance and the possibility of bacterial mutation in a high-stress environment [29].

This review focuses on the potential of marine actinobacteria to produce compounds with antibacterial, antifungal (against some fungi such as *Candida albicans* and *Aspergillus fumigatus*) [30,31,32,33], antibiofilm activity, and QQ, [34] which inhibits or disrupts an important chemical communication system in bacteria. This involves pathogenic gene expression and metabolism regulation in response to the density of bacterial populations through the production and sensing of some small signal molecules called auto-inductors, both in the same species (intraspecies) as well as among different species (interspecies) [30].

Of the biological activities studied, the antibacterial activity was the most frequently reported in the articles included, with a prevalence of 64% approximately (as shown in Supplementary Table S2), providing the minimum inhibitory concentration (MIC) in some cases (as is presented in Table 1).

**Figure 5:**
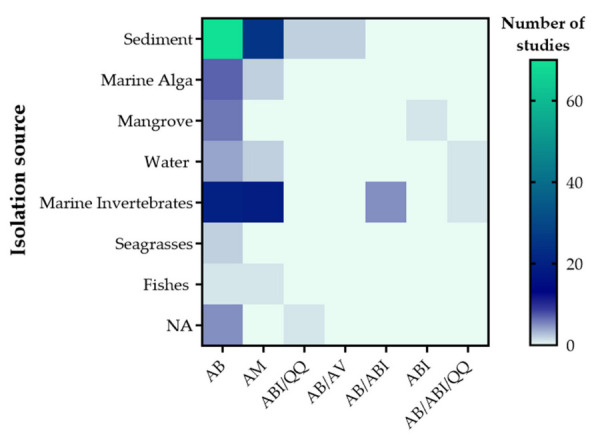
Heatmap of the number of articles included in this study that reported the isolation source of compounds with the biological activity of interest. Isolation sources are arranged from top to bottom, starting with the largest number at the top left. Bioactivities are shown at the bottom from left to right by the largest number of papers reported. The color bar represents the number of studies that reported the source of isolation of bioactive metabolites, from white to blue (lower values), blue to green (medium values), and green (high values). AB: antibacterial activity; AM: antimicrobial activity (activity against bacteria, fungus, parasites); ABI/QQ: antibiofilm and QQ activity; AB/AV: antibacterial and antiviral activity; AB/ABI: antibacterial and antibiofilm activity; ABI: antibiofilm; AB/ABI/QQ: antibacterial, antibiofilm and QQ activity.

Furthermore, this activity was mostly found in compounds obtained from actinobacteria isolated from sediments and marine invertebrates. Likewise, antibiofilm activity and QQ were reported in bacteria of these two sources, and also found in compounds isolated from seawater and mangroves, water actinobacteria, as shown in Figure 5.

Table 1 and Table 2 shows the antibacterial and antimicrobial activity, respectively, of crude extract or compounds, obtained from marine actinobacteria expressed in MIC, in which compounds/extracts/fractions with MICs from 0.01–0.02 up to 100, 128, 256, 500 and 1000 μg/mL are reported. Compounds that present activity through the inhibition zone are shown in Tables S3 and S4. Likewise, Table 1 and Table 2 present the pathogenic bacteria’s target. It is evident that the actinobacteria metabolites exhibit activity towards two of three interesting bacteria that are related to the development of acne vulgaris disease, including MRSA (methicillin-resistant *Staphylococcus aureus*), *S. aureus*, *S. epidermidis* and MRSE (methicillin-resistant *Staphylococcus epidermidis*), but not against *C. acnes*.

**Table 1:** Antibacterial capacity of actinobacterial crude extracts or compounds.

Genus	Pathogen Target	Compounds/Extracts	MIC (μg/mL)	Ref.
*Streptomyces* sp.	MRSA ^1^	Napyradiomycins 1	0.016	[35]
		Napyradiomycins 8	0.002	[35]
*Streptomyces* sp.	MRSA ^1^	Marinopyrrole A	3.24	[36]
		Marinopyrrole B	3.24	[36]
*Streptomyces* sp.	*S aureus* ATCC NR-46171	4-methoxyacetanilide	32.4	[18]
*Streptomyces* sp.	*S. aureus*	Flaviogeranin D	9.2	[37]
		Flaviogeranin C2	8.1	[37]
*Streptomyces* sp.	*S. aureus*	1-hydroxy-1-norresistomycin	>40	[38]
*Streptomyces* sp.	MRSA ^1^	Fridamycin A	500	[23]
		Fridamycin D	62.5	[23]
*Streptomyces* sp.	MRSA ^1^	Chromomycin A_3_	0.698	[39]
*Streptomyces* sp.	MRSA ^1^	Extract	2	[40]
*Streptomyces* sp.	MRSA ATCC 33591	Actinomycins D_1_	0.125	[41]
		Actinomycins D_2_	0.25	[41]
		Actinomycins D_3_	0.5	[41]
		Actinomycins D_4_	0.25	[41]
		Actinomycins D	0.25	[41]
*Streptomyces* sp.	*S. aureus* CCARM 3090	Grincamycin L	6.25	[42]
*Streptomyces* sp.	MRSA ^1^	Compound ^2^	2	[43]
*Streptomyces* sp.	*S. aureus* (ATCC 29213)	2,4-dichloro-5-sulfamoyl benzoic acid	0.8–4	[44]
*Streptomyces* sp.	*S. aureus* (ATCC 25923).	Dionemycin	0.5–2	[45]
*Streptomyces* sp.	*S. aureus* ATCC 43300	Extract	7.9	[46]
*Streptomyces* sp.	*S. aureus* ATCC 43300	Extract	12.5	[47]
	*S. epidermidis* (ATCC 12228)	Extract	25	[47]
*Streptomyces* sp.	*S. aureus*	Aborycin	8.0~64	[48]
	MRSA ^1^		16~64	[48]
	MRSE ^3^		128	[48]
*Streptomyces* sp.	MRSA ^1^	Supernatant	0.78	[49]
*Streptomyces* sp.	MRSE ^3^	Dehydroxyaquayamycin	16.0	[50]
*Streptomyces* sp.	MRSA ^1^	Medermycin	2	[51]
		G15-F	4	[51]
*Streptomyces* sp.	MRSA ^1^ ATCC BAA-44	Bisanhydroaklavinone	6.25	[19]
		1-Hydroxybisanhydroaklavinone	50	[19]
*Streptomyces* sp.	MRSA ^1^	11′,12′-dehydroelaiophylin	1−4	[52]
	MRSA ^1,^ MRSE ^3^	Elaiophylin	1−4	[52]
		11-monomethoxylated derivative	2−16	[52]
		Compound 6 ^4^	2−16	[52]
*Streptomyces* sp.	MRSA ^1^	Lactoquinomycin A	0.25–0.5	[53]
	MRSA ^1^	Stremycin A	16	[54]
		Stremycin B	16	[54]
*Streptomyces* sp.	MRSA ^1^	Quinomycin G	16–64	[55]
	MRSE ^3^			
	MSSE ^5^			
*Streptomyces* sp.	*S. aureus* (ATCC 6538)	Actinomycins X2	0.394	[56]
	MRSA ^1^ (ATCC 43300)	Actinomycins X2	0.190	[56]
	*S. aureus* (ATCC 6538)	Actinomycins D	0.389	[56]
	MRSA ^1^ (ATCC 43300)	Actinomycins D	0.188	[56]
*Streptomyces* sp.	MRSA ^1^	Extract ^7^	6.25	[25]
	MSSA ^6^	Extract ^7^	12.5	[25]
*Streptomyces* sp.	MRSA ^1^	Extract ^8^	12.5	[25]
	MSSA ^6^			
*Streptomyces* sp.	MRSA ^1^	Borrelidins J	0.195	[28]
*Streptomyces* sp.	*S. aureus*	Extract	256	[57]
*Streptomyces* sp.	*S. epidermidis*	Extract	128	[57]
*Streptomyces* sp.	MRSA ^1^	Streptopertusacin A	40	[58]
		21,22-en-bafilomycin D	12.5	[58]
		21,22-en-9-		
		hydroxybafilomycin D	12.5	[58]
*Streptomyces* sp.	*S. aureus*	Lobophorins E	32	[59]
	ATCC 29213	Lobophorins F	8	[59]
*Streptomyces* sp.	MRSA ^1^	Pyrrole-derivative	2.8	[60]
*Streptomyces* sp.	MRSA ^1^	Julichromes Q_11_	16–64	[61]
	*S. aureus* ATCC 29213	Julichromes Q_10_	16–64	[61]
		Julichromes Q_6.6_	16–64	[61]
		Julichromes Q_6_	16–64	[61]
*Streptomyces* sp.	MRSA ^1^, *S. aureus*	Lobophorin-like spirotetronate	64	[62]
*Streptomyces* sp.	MRSA ^1^, *S. aureus*	Ansamycins	32	[63]
	MRSA ^1^	(-)-Streptophenazine B	4.2	[63]
*Streptomyces* sp.	MRSA ^1^	Neo-actinomycin A	16–64	[64]
*Streptomyces* sp.	*S. aureus* ATCC 29213	MarfuraquinocinsA	8.0	[65]
*Streptomyces* sp.	MRSE ^3^ shhs-E1	Marfuraquinocins C	8.0	[65]
	*S. aureus* ATCC 29213	Marfuraquinocins D	8.0	[65]
*Streptomyces* sp.	MRSA ^1^ ATCC 43300	7,8-dideoxygriseorhodin C	0.08–0.12	[66]
		Oxacillin and 7,8-dideoxygriseorhodin C	0.01–0.02	[66]
*Streptomyces* sp.	MSSA ^6^ 11497	Desertomycin G	4.0	[67]
	MRSA ^1^ ATCC 43300	Desertomycin G	4.0	[67]
	MRSA ^1^ ATCC 25923	Desertomycin G	4.0	[67]
*Streptomyces* sp.	*S. aureus* ATCC 6518, MTCC 3160	Aromatic polyketide	32.40	[68]
	MRSA ^1^			
*Streptomyces* sp.	*S. aureus* ATCC 29213	Napyradiomycins 1–8 ^9^	0.5 to 32	[69]
	MRSA ^1^			
*Streptomyces* sp.	*S. aureus* ATCC 29213	Marinopyrroles A–C	<1	[70]
		Marinopyrroles F	3.1	[70]
*Streptomyces* sp.	MRSA ^1^-ATCC33591	A80915A ^10^	1–4	[71]
*Streptomyces* sp.	MRSA ^1^ ATCC 43300	Polyketide 13 ^11^	2	[72]
*Streptomyces* sp.	MRSA ^1^	Fijimycins A–C Etamycin A	4–16	[73]
*Streptomyces* sp.	*S. aureus* HA- and CA-	Etamycin	1–2	[74]
*Streptomyces* sp.	MRSA ^1^	Lydicamycin congeners	1.56–12.5	[75]
*Streptomyces* sp.	MRSA ^1^	Salinamide F	100	[76]
	*S. aureus* (ATCC 12600)			
*Streptomyces* sp.	*S. aureus*	Antimycin B1	32	[77]
*Streptomyces* sp.	*S. aureus*	Merochlorins G	16	[78]
		Merochlorins J	2	[78]
*Streptomyces* sp.	*S. aureus*	cyclo(L-Pro-L-Tyr)	160	[79]
		cyclo(L-Pro-L-Phe)	180	[79]
*Streptomyces* sp.	MRSA ^1^	Actinomycin X2	3.125–12.5	[80]
		Actinomycin D	12.5–25	[80]
*Streptomyces* sp.	*S. aureus*	1,3-Benzodioxole	256	[81]
*Streptomyces* sp.	*S. aureus ATCC* 29213	Desotamide, Desotamide B	16	[82]
	MRSE		32	[82]
*Streptomyces* sp.	*S. epidermidis*	Streptophenazines G	3.68	[83]
		Streptophenazines F	6.77	[83]
*Streptomyces* sp.	MRSA ^1^	Citreamicin θ A	0.25	[84]
	ATCC43300	Citreamicin θ B	0.25	[84]
		Citreaglycon A	8.0	[84]
	*S. aureus* UST950701-005	Dehydrocitreaglycon A	16	[84]
*Streptomyces* sp.	*S. aureus* DSM346	Alageninthiocin	15	[85]
		Geninthiocin	4	[85]
		Val-geninthiocin	8	[85]
		Indolocarbazole staurosporine	19	[85]
*Streptomyces* sp.	MRSA ^1^	Anthraquinone derivatives	6.25	[86]
*Streptomyces* sp.	MRSA ^1^	Extract	1000	[87]
*Streptomyces* sp.	*S. aureus*	Extracts	312–2.5 × 10^2^	[88]
*Streptomyces* sp.	*S. aureus*	Extract	400	[89]
*Streptomyces* sp.	*S. aureus*	Extract AIA12	2.5 × 10^2^	[90]
	ATCC 25923			
		Extract AIA17	310	[90]
*Streptomyces* sp.	MRSA ^1^	1-Acetyl-β-Carbonile	128–256	[91]
	MSSA ^6^	1-Acetyl-β-Carbonile	64	[91]
*Streptomyces* sp.	MRSA ^1^	Chlororesistoflavins A	0.25	[92]
	MRSA ^1^	Chlororesistoflavins B	2.0	[92]
*Streptomyces* sp.	*S. aureus*	Ligiamycin A	16	[26]
	*S. aureus*	Ligiamycin B	64	[26]
*Verrucosispora* sp.	*S. aureus ATCC* 33591	Active fraction	16–32	[93]
*Verrucosispora* sp	*S. aureus* ATCC29213	Proximicins B	16	[94]
	MRSA shhs-A1			
*Verrucosispora* sp.	MRSA ^1^	1-hydroxy-2,5-dimethyl benzoate	12.5	[95]
*Verrucosispora* sp.	MRSA ^1^	Proximicin B	3.125	[95]
*Micromonospora* sp.	*S. aureus* ATCC 29213	Kendomycins B	0.5–2	[96]
	*S. aureus* 745524	Kendomycins C	0.5–1	[96]
	MRSA ^1^ shhs-A1	Kendomycins D	1–4	[96]
*Micromonospora* sp.	MRSA ^1^	2-ethylhexyl 1H-imidazole-4- carboxylate	16	[97]
*Micromonospora* sp.	*S. aureus* ATCC 29213	Micromonohalimanes B	40	[98]
*Micromonospora* sp.	*S. aureus* ATCC 29213	Rabelomycin	1	[99]
		Phenanthroviridone	0.25	[99]
*Micromonospora* sp.	*S. aureus* ATCC 29213	homo-dehydrorabelomycin E	1	[100]
*Nocardiopsis* sp.	MRSA ^1^	Bis (2-ethylhexyl) phthalate	7.81	[101]
	MRSA ^1^	4-bromophenol	15.62	[101]
	ATCC NR-46071			
*Nocardiopsis* sp.	MRSA ^1^	Nocardiopsistin A	12.5	[102]
		Nocardiopsistin B	3.12	[102]
		Nocardiopsistin C	12.5	[102]
*Nocardiopsis* sp.	MRSA ^1^	α-Pyrone	12.5	[103]
*Nocardiopsis* sp.	MRSA ^1^	Extracts	115–125	[104]
*Marinispora* sp.	MSSA ^6^	Lipoxazolidinone A	1–2	[105]
	MRSA ^1^			
*Marinispora* sp.	MRSA ^1^	Lynamicins A–E	2.2–45	[106]
	MRSE ^3^ ATCC 700578c			
*Pseudonocardia carboxydivorans*	*S. aureus* ATCC 6538P	Branimycins C	32	[107]
	MRSA ^1^ MB5393	Branimycins C	20–40	[107]
*Kocuria* sp.	MRSA ^1^ ATCC 43300-	Kocurin	0.25–0.5	[108]
*Solwaraspora* sp.	MRSA ^1^	Solwaric acids A	32	[109]
		Solwaric acids B	32	[109]
	MSSA ^6^	Solwaric acids A	64	[109]
		Solwaric acids B	64	[109]
*Salinispora* sp.	MRSA ^1^	Rifamycin W	15.62	[110]

^1^ MRSA: Methicillin-resistant *Staphylococcus aureus.* ^2^ Compound: 1 [2-hydroxy-5-((6-hydroxy-4-oxo-4Hpyran-2-yl) methyl)-2-propylchroman-4-one]. ^3^ MRSE: Methicillin-resistant *Staphylococcus epidermidis.* ^4^ Compound 6: Compound name no reported. ^5^ MSSE: Methicillin-susceptible *Staphylococcus epidermidis.* ^6^ MSSA: Methicillin-susceptible *Staphylococcus aureus.* ^7^ Extract: Extract Co-culture (MRSA). ^8^ Extract: Extract Co-culture (*Pseudomonas aeruginosa*). ^9^ Napyradiomycins 1–8: Except compound 3. ^10^ A80915A: Napyradiomycin derivatives. ^11^ Polyketide 13: [=2-hydroxy-5-((6-hydroxy-4-oxo-4H-pyran-2-yl) methyl)-2- propylchroman-4-one].

It is well known that actinobacteria are a phylum with the potential to produce molecules with innumerable bioactivities and with multiple applications. Santos et al. [33] studied actinobacteria strains isolated from a marine sponge, in which antimicrobial activity was previously reported (due to this it is not described in Table 2) against MRSA (methicillin-resistant *S. aureus* MB 5393), which could also be involved in skin infections, as well as the fungus *Aspergillus fumigatus* ATCC46645, demonstrating that it also had the capacity to induced lipid reduction on the larvae of zebrafish [33], revealing its potential use in anti-obesity treatments.

Other compounds or strains were reported with activity, but this was presented in growth inhibition percentage for Aa3_DN216_4B10_1, which showed a significant growth inhibition (61%) against MRSA [22].

A wide variety of compounds with antimicrobial activity were reported in plants, such as flavonoids. Interestingly, in this systematic review, some studies reported flavonoids from sponge-derived actinobacteria. Flavonoids are a group of natural substances with variable phenolic structures; they are found in fruits, vegetables, grains, bark, roots, stems, flowers, and wine. These are an important class of natural products; particularly, they belong to a class of plant secondary metabolites having a polyphenolic structure [84].

Historically, flavonoids have been recognized with a broad spectrum of health-promoting effects because of their antioxidative, anti-inflammatory, anti-mutagenic, and anti-carcinogenic properties with their application in various diseases such as cancer, Alzheimer’s disease (AD), atherosclerosis, etc. [131]. Cao et al. [118] reported two new lavandulylated flavonoids, 6-lavandulyl-7-methoxy-5,20,40-trihydroxylflavanone and 50-lavandulyl-40-methoxy-2,4,20,60-tetrahydroxylchalcone (Table 2), which had a broad-spectrum of antimicrobial activity against both Gram-positive and Gram-negative bacteria and fungus such as *Candida albicans* [118]. Other compounds with antibacterial activity included in this systematic review are citreamicins, which are polycyclic xanthones (belong to flavonoids class) obtained from marine-derived *Streptomyces caelestis*, isolated in the coastal water of the Red Sea [84]. This *S. caelestis* showed antibacterial activity against a variety of Gram-positive bacteria, including MRSA and vancomycin-resistant *Enterococcus faecalis* (VRE). Four compounds were isolated from *S. caelestis* (Table 2), with antibacterial activity against *S. aureus* UST950701-005 with a MIC from 1 to 16 µg mL^−1^ and three had antibacterial activity against MRSA with a range of MIC between 0.25 and 8 µg mL^−1^ [84].

On the other hand, anthracycline compounds with antibacterial and antimicrobial activity have also been reported among the metabolites derived from marine actinobacteria. Anthracyclines are known as an important class of anticancer compounds used for many years in the treatment of leukemia, breast carcinoma, and other solid tumors. However, their application in cancer treatment has been decreased due to their toxic, dose-related side effects such as stomatitis, gastrointestinal disorders, and cumulative cardiotoxicity. Anthracyclines belong to the group of tetramic acids and have been reported to have antibacterial activity toward Gram-positive bacteria such as vancomycin-resistant *Enterococcus* (VRE). Cong et al. discovered novel anticancer and anti-infective natural products from marine *Streptomyces* sp. SCSIO 41399 which were isolated from coral *Porites* sp. These compounds were isotirandamycin B and two known tirandamycin derivatives. This study is one of the two that in this systematic review that reported a coral with anti-infective activity toward *Streptococcus agalactiae* and *S. aureus,* which may be useful in the control of acne-related bacteria [119].

Other compounds reported with antimicrobial activity in this systematic review were Chromomycins, Napyradiomycins, Marinomycins, and Kokumarin.

Chromomycins are members of the aureolic acid family, and they are polyketides with a tricyclic aglycone core with two aliphatic side chains at C-3 and C-7 and two sugar chains at C-2 and C-6, similar to other aureolic acid family members. Chromomycins interact with the DNA helix minor groove in regions with high GC (guanine–cytosine) content and in a non-intercalative way with Mg^2+^ cations, causing DNA damage in treated cells [121].

Napyradiomycins (NPDs) for their part constitute an interesting family of halogenated natural compounds NPDs that consist of a naphthoquinone core, a prenyl unit attached at C-4a, a monoterpenoid substituent at C-10a, and some congeners have a methyl group at C-7 [123].

Marinomycins possess unique polyene–polyol structures and have unique photoreactivities and chiroptical properties [129]. Finally, Kokumarin was the only compound reported to have antimicrobial activity against MRSA isolated from skin infections [128].

In addition to the antibacterial and antimicrobial activity exhibited by extracts or isolated compounds of marine actinobacteria recovered in this systematic review, some bacteria, especially of the *Streptomyces* and *Nocardiopsis* genera, have been shown to have more than one biological activity such as both antibacterial and antibiofilm activity, as shown in Table 3, with high activity against *S. aureus* and methicillin-resistant *S. epidermidis,* related with the development of acne vulgaris.

Four of five compounds reported to have antibacterial and antibiofilm activity were isolated from the *Streptomyces* genus and these activities were reported against MRSA; only one unidentified compound had an effect against MRSE. This compound, named SKC3, exhibited an antagonistic effect against growth and biofilm formation of the methicillin-resistant *S. epidermidis* at a concentration below the MICs (Table 3). Interestingly, the biofilm inhibitory concentration (BIC_90_) of SKC3 was 3.95 μg/mL, and this had no considerable influence on bacterial growth. In addition, SKC3 also had an effect in inhibiting the growth and biofilm formation of other strains such as MSSA, MRSA, and VRSA, however, was ineffective against the tested Gram-negative *P. aeruginosa* strains [133].

The compound PVI331 had a prominent antibacterial activity with a MIC of 1 μg/mL, (Table 3) and showed biofilm inhibition at a 92.17 ± 1.67% at 4 μg/mL, concentration against MRSA and it was more effective than the anti-MRSA antibiotic vancomycin, which was used at a concentration of 8 mg/mL, and the biofilm inhibition was 32.58 ± 2.52% [132].

8-O-metyltetrangomycin is an angucycline antibiotic that showed a significant antibiofilm activity toward MRSA, ranging from 52.85 to 86.64% inhibition. Similar to compound PVI331, this angucycline compound exhibited more antibiofilm potential than vancomycin and the highest range of inhibition was observed at 4× MIC, suggesting the stronger potential to reduce biofilm formation that possesses these compounds [10].

Compounds PVI401 and PVI402 exhibited antibacterial activity against MRSA, (Table 3), however, only PVI401 showed antibiofilm activity toward *S. aureus* ATCC25923; this effect was dependent on the concentration obtained in the antibacterial assay of compound PVI401, with poor biofilm formation when compared to controls when the pathogen was treated with a 4× MIC concentration at 2 μg/mL, of PVI401 [134].

Regarding antibiofilm activity, the same compounds were reported by Hifnawy et al. [27] to have antibacterial activity against Gram-negative and Gram-positive bacteria and antibiofilm activity, and the compounds tubermycin and p-anisamide had potent antibiofilm activity against *P. aeruginosa* with inhibition rates of 94 and 73% respectively. On the contrary, compounds **1**, **2** and **9** had antibiofilm activity against *E. coli* with inhibition ranges of 34–54%, and only Compounds **1** and **2** showed a potent to moderate inhibition against *S. aureus* with a percentage of inhibition rates of 50 and 75% respectively [27].

Concerning antibacterial, antibiofilm activity, and QQ, only 2 of 177 papers described compounds with three activities and two more articles reported antibiofilm and QQ (Table 4). These papers evaluated the QQ ability of actinobacteria-derived metabolites. All studies evaluated the AI-1 (Autoinducer 1) system of quorum sensing, using the reporter strain, *Cromobacterium violaceum*. This strain produces a visible purple pigment called violacein, which is under positive regulation by the N-acyl-homoserine lactone CviI/R quorum-sensing system. This system has been reported in Gram-negative bacteria mainly [135,136,137]. Moreover, one of the studies also evaluated the inhibition of the LuxS/AI-2 quorum-sensing system. In this system, the signal molecule is regulated by the *luxS* gene [138] and it has been reported that is utilized by more than 40 species of Gram-positive and Gram-negative bacteria for communication and transmission [136]. This system has been reported in *C. acnes*, *S. aureus,* and *S. epidermidis*, bacteria under this study, nonetheless, both *S. epidermidis* and *S. aureus* also have been reported to use peptides autoinducers (AIP), regulated by *agr* system for quorum sensing [139]. Nevertheless, any article included in this systematic review that reported inhibition in this system could further be investigated as an effective treatment of acne vulgaris.

From these investigations, two compounds are described as having antibacterial and antibiofilm activity, as well as QQ: one of these is butenolide, which is a compound isolated from marine actinobacteria with antifouling activity studied previously; this compound inhibited quorum sensing and is an unspecific inhibitor due to having the ability to inhibit the AHL system through the inhibition of the violet pigment of two *C. violaceum* strains, *CV026* (short-chain AHLs) and VIR24 (long-chain AHLs), inhibiting short-chain AHLs at a concentration of 100 μg/mL and long-chain AHLs at 50 μg/mL, and with growth inhibition being observed at concentrations of 25–50 μg/mL. This compound also inhibits the AI-2 system through bioluminescence of indicator strains *Vibrio harveyi* BB170, at concentrations of 5, 12.5, and 25 μg/mL with a reduction of luminescence of ~25, ~50, and over 70%, respectively. However, at concentrations above 12.5 μg/mL, it caused growth inhibition against the bacterial cells (Table 4) [138]. Despite this, it is considered to have low antibacterial activity against diverse types of pathogens (both Gram-positive and Gram-negative bacteria) [138].

Likewise, it was found that butenolide not only effectively inhibited the biofilm formation but also eradicated pre-formed biofilms of tested bacteria and it also had a synergistic effect with tetracycline; it was a potential tetracycline enhancer against biofilm-associated infection-producing bacteria such as *E. coli, P. aeruginosa*, and MRSA [138].

Another compound with antibacterial, antibiofilm, and QQ activity is a melanin pigment. It was discovered from marine *Nocardiopsis* sp., which exhibited antibacterial activity toward *Bacillus* sp. from extract JN2 with growth inhibition of 68 and >40% against *S. aureus* at a concentration of 150 μg mL^−1^. Respecting its antibiofilm activity, both the pigments (JN1M and JN2M) inhibited the growth of quorum-sensing bacteria *C. violaceum* MTCC 2656 (Table 4) [137].

**Table 4:** Anti-biofilm, antibacterial, and quorum-quenching activity of crude extracts or compounds from marine actinobacterial.

Genus	Target Bacteria in Antibiofilm Activity	MBIC ^1^	Compounds/ Extracts	Percentage Decreased Biofilm	QS System	QQ Activity (IC50)	Biosensor Strain	Ref.
*Streptomyces* sp.	MRSA ^2^	200	Butenolide	>70	AI-2 up to 70%	NA ^3^	*Vibrio harveyi BB170*	[138]
					AHL inhibition up to 97%		*C. violaceum*	[138]
	*S. aureus*	100	Extract	78.9	AHL	NA ^3^	*C. violaceum* 12472	[140]
*Nocardiopsis* sp.	*S. aureus*	NA ^3^	Melanin JN1M	64.2	AHL	NA ^3^	*C. violaceum MTCC 26563*	[137]
			Melanin JN2M	65.9	AHL	NA ^3^	*C. violaceum MTCC 26563*	[137]
*Nocardiopsis* sp.	*S. aureus*	20 vol % ^4^	Culture liquid of JS106	77.94	AHL	NA ^3^	*C. violaceum* 12472	[29]
	NA ^3^	NA ^3^	Questiomycin A	NA ^3^	AHL	6.82	*C. violaceum* 12472	[29]
	NA ^3^	NA ^3^	2-hydroxyacetate-3-hydroxyacetamido-phenoxazine (HHP)	NA ^3^	AHL	23.59	*C. violaceum* 12472	[29]

^1^ MBIC: The minimum biofilm inhibitory concentration. ^2^ MRSA: Methicillin-resistant *Staphylococcus aureus*. ^2^ MRSE: Methicillin-resistant *Staphylococcus epidermidis*. ^3^ NA: Information not reported. ^4^ 20 vol %: Concentration expressed in percentage.

In addition, there are compounds reported with antibiofilm and QQ activity. The liquid culture and crude extract of *Nocardiopsis* sp. displayed a decreased antibiofilm activity against *S. aureus* and QQ by inhibiting the violacein production of strain *C. violaceum* 12472, respectively. Likewise, the compounds Questiomycin A and 2-hydroxyacetate-3-hydroxyacetam-ido-phenoxazine (HHP) isolated from this liquid culture also showed QQ activity against *C. violaceum* 12472 at a concentration of 40 μg/mL (Table 4). This compound belongs to the phenoxazinones group and is a structurally unique natural product containing a tricyclic core heterocyclized by nitrogen and oxygen atoms [29].

### 2.5. Actinobacteria Producing Quorum Quenching Metabolites

Regarding the QQ activity, it was evaluated in only 2.8% of the papers, and the mechanism of inhibition used was the AHL (acyl-homoserine lactone) autoinducer (AI-1), through the indicator strain *C. violaceum*; one only study reported the effect of the extract of marine actinobacteria against mechanism two, the LuxS enzyme autoinducers 2 (AI-2), through the bioluminescence of *V. harveyi* BB170 [138]. Table 5 shows the papers with quorum-quenching activity.

### 2.6. Strategies to Maximize Anti-Infective Metabolites Activity and Yield

#### 2.6.1. Culture Conditions to Anti-Infective Production Metabolites

Actinobacteria fermentations often do not generate a high yield of active compounds [51]. It is well known that the culture conditions significantly affect bacterial metabolism. Likewise, the composition of the culture medium is related to the metabolic capacities of the producing organism, influencing the biosynthesis of antibiotics [114]. Some studies included in this systematic review (48 of 177) carried out the identification of the variables that are related to the increase in the production of compounds with anti-infective activity, through some biostatistical methods such as the Placket–Burman design and the response surface method [60]. These analyses revealed that carbon and nitrogen sources played a key role, with the nitrogen source in some cases being more prominent [142] in addition to pH, temperature, and agitation speed.

**Figure 6:**
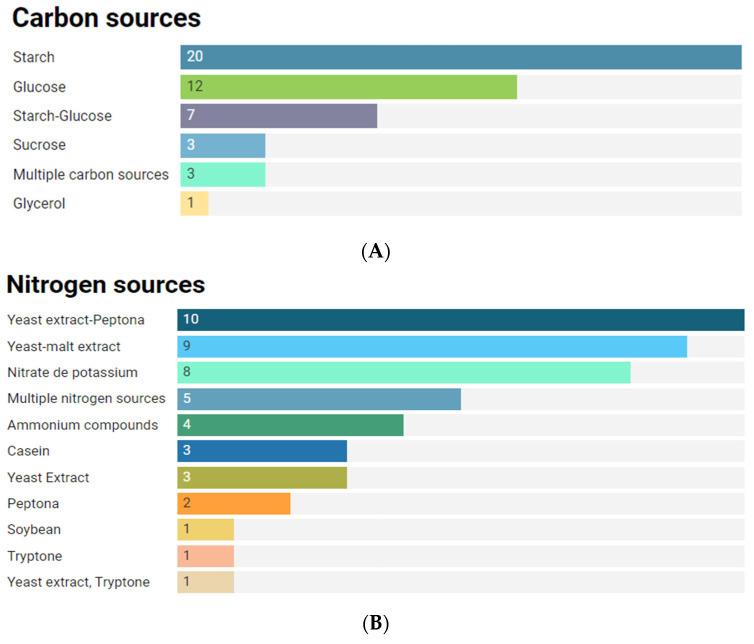
(**A**). Most used carbon sources to maximize the anti-infective compound. (**B**). Most used nitrogen sources to maximize the anti-infective compounds. Only 48 of 177 papers reported culture conditions.

Starch was described as a carbon source used to achieve maximum production of the anti-infective compound as reported by Djini et al. [43] as presented in Figure 6A. Likewise, Norouzi et al. revealed a significant effect of starch in combination with Peptone (as nitrogen source) and pH, and calcium carbonate, reaching up to a 218% increase in production yield of anti-MRSA compounds [60], and Mohamedin et al. reported antagonistic activity produced from the optimized culture conditions against multidrug-resistant *Staphylococcus epidermidis,* which showed about a 1.37-fold increase using starch as the carbon source and potassium nitrate and yeast extract as the nitrogen source [143].

Other carbon sources reported to increase the production of compounds with anti-infective potential were glucose [18,27,33], sucrose [37,132,144] starch–glucose [23,31,94] among others.

Regarding the nitrogen source, the most common were yeast extract–peptone [81,94,102], yeast–malt extract [20,38,145], potassium nitrate [10,40,146], ammonium compounds as ammonium sulfate [147], ammonium chloride [79], ammonium nitrate [114], and casein [22,43,148] as shown in Figure 6B. The quenching potential also has been subjected to optimization processes to maximize its performance, finding that soybean meal and sodium chloride were two crucial factors in the culture medium that significantly increased both the bioactivity and metabolite production (302 and 241%, respectively) when compared to the original condition [29].

Also, some studies highlighted the need for seawater not only for the cultivation of the strains, but also to produce antibiotics [43,105,149], making it clear that this depends on the concentration of salt [142,149]. Figure 6 presents the carbon and nitrogen sources most used in the rise of anti-infective compound production.

On the other hand, Xu et al., reported that the supplementation of the rare earth salt Lanthanum chloride (LaCl3) during fermentation of HB-J378 significantly increased the yield of these angucyclines [102]. This similarly occurred with the strains N816 and S355 isolated from marine sponge actinomycetes, which showed potent anti-MRSA activity elicited due to the addition of LaCl3 that was significantly enhanced in the J378 strain, which shows LaCl3 to be an effective elicitor [102].

#### 2.6.2. Co-Culture Combination as Strategies to Maximize Anti-Infective Metabolites in Marine Actinobacteria

The co-culture of microbial strains can activate the production of compounds that in monoculture are not obtained or the accumulation of metabolites is less. In addition, it has been considered that this strategy also contributes to activating silent biosynthetic gene clusters, leading to the improved production of natural compounds that do not occur under laboratory conditions [86]. In the marine environments, bacterial secondary metabolites production usually depends on their interactions with other microbes or is regulated by environmental or stressing conditions such as competition for nutrients or space [27,86,150].

There are diverse ways to have a microbial strain co-culture; one of the most common is between fungus and bacteria as was reported in the microbial co-culture combination of a sponge-derived actinomycete *Streptomyces rochei* MB037 and a gorgonian-derived fungus *Rhinocladiella similis,* which induced the production of related polyketides and exhibited significant antibacterial activity against methicillin-resistant *S. aureus* with a MIC value of 0.195 mg/mL [28]. Furthermore, another way of co-culture is the co-cultivation between bacteria of different or the same genus, such as the co-culture of two red marine sponge-associated actinomycetes *Micromonospora* sp. UR56 and *Actinokinespora* sp. EG49, which induced the accumulation of metabolites with antibacterial and antibiofilm activity, that were not traced in their axenic cultures [27]. The compounds belong to the phenazine class and have been isolated and characterized previously. In total, authors obtained five compounds; from them, Compounds 1 (dimethyl phenazine-1,6-dicarboxylate), 2 (phencomycin), and 9 (*N*-(2-hydroxyphenyl)-acetamide) showed considerable antibacterial activity against *S. aureus* with growth inhibitions of 47, 69, and 53% respectively. In addition, Compounds 3 (tubermycin) and 10 (p-anisamide) displayed potent antibacterial activity against *P. aeruginosa* with growth inhibition of 94 and 70% respectively [27]. Also, the co-culture between marine-derived actinobacteria and human pathogens in this systematic review has been reported, which resulted in increased production of three antibiotics: granaticin, granatomycin D, and dihydrogranaticin B, and it also strongly enhanced biological activity against the Gram-positive human pathogens such as MRSA [25].

### 2.7. Main Families of Compounds Found in Marine Actinobacteria with Antibacterial Activity

An enormous variety of compounds were reported in the papers included in this systematic review; these have been arranged considering the type of activity that they exhibited and grouped in families.

Among families, polyketides were the most reported; these types of compounds are a vast variety of constituents and represent a highly diverse structural class of products, demonstrating varied biological functions [72]. Polyketides are secondary metabolites produced from bacteria, fungi, plants, and animals, and bacteria from the *Streptomyces* genus, which are thought one of the polyketides producers [28]. Polyketides are made up of many compounds, including macrolides, reported in 7 of 177 papers, aromatic polyketides in 9 of 177 (including angucyclines), and so on.

Table 6 displays the family compounds, their constituents, and the frequency that were presented.

Macrolides are a class of antibiotics derived from *Saccharopolyspora erythraea* (originally called *Streptomyces erythreus*), a type of soil-borne bacteria. They are bacteriostatic antibiotics in that they suppress or inhibit bacterial growth rather than killing bacteria completely and possess a macrocyclic lactone ring containing eight or more atoms, and polyketide [161]. They act by inhibiting the protein synthesis of bacteria by binding to the 50S ribosomal element [162].

Macrolides, especially erythromycin together with clindamycin, which is a lincosamide (isolated from an actinobacterium, *Streptomyces lincolnensis* obtained from the soil in the region of Lincoln, Nebraska, United States), are the main antibiotics recommended as the first-line therapy in the acute inflammatory phase of acne [163].

Both have similar mechanisms of action, and lincosamides have even been integrated with macrolides in a group called “macrolides and similar” [164].

The angucycline group of antibiotics and aromatic polyketide natural products belong to a specific group of polycyclic aromatic polyketides, which exhibit anticancer and antimicrobial activities [165]. This type of antibiotic was first discovered as a tetrangomycin isolated from *Streptomyces rimosus* in 1965. Members of angucyclines are characterized by an angular tetracyclic (benz[α]anthracene) structure with a hydrolyzable sugar moiety and they are biosynthesized by type II polyketide synthases (PKSs) via decarboxylative condensations of a short acyl-CoA starter and nine extender units [146,165]. *Streptomyces* sp. is known as the major producer of angucyclines [54].

Aromatic polyketides, representative substances of type II polyketides, have significant therapeutic properties, including tetracycline and anthracycline-type doxorubicin, which are typical of aromatic polyketides with pharmacological applications [53].

Flavonoid structures are characterized by a 15-carbon skeleton, in two aromatic ring systems (A, and B rings) and a heterocyclic ring C, the ring containing embedded oxygen [166]. This carbon structure can be abbreviated as C6–C3–C6 rings and with different substitution patterns to produce a series of subclass compounds, such as flavones and flavonols, as the quercetin, isoflavones, etc. [166]. Nevertheless, there are other flavonoids without a C6–C3–C6 skeleton, for instance, biflavones, furan chromones, and xanthones [166].

Another family of compounds reported to have antibacterial activity are phenazines; these are heterocyclic nitrogenous compounds that consist of two benzene rings attached through two nitrogen atoms and substituted at different sites of the core ring system. They have been isolated in substantial amounts from terrestrial bacteria such as *Pseudomonas*, *Streptomyces*, and other genera from marine habitats [27]. Based on earlier reports on the biological activities of this class of compounds, it was suggested both DNA gyrase B (Gyr-B) and pyruvate kinase (PK) were the possible molecular targets of their antibacterial activity [27].

Chromopeptide lactone antibiotics is another family of compounds among which actinomycins are one of their constituents; actinomycin D is one of the older anticancer drugs and has been studied extensively and widely used clinically for the treatment of several types of malignant tumors. Despite their initial discovery more than 70 years ago, actinomycins continue to be a focus of many research areas, especially in their biological activity and medicinal use [116].

### 2.8. Main Family Compounds Found in Marine Actinobacteria with QQ Activity

The inhibition of quorum sensing is a therapeutic target for the treatment of diseases generated by bacteria that has gradually been gaining interest, since to date, there have been no reports of the development of resistance by bacteria against this mechanism. Few studies to date have reported compounds isolated from marine actinobacteria with the ability to inhibit quorum sensing; however, some families of compounds that have exhibited this activity have already been identified. Among these, fatty acyl compounds, phenoxazines, lactones, and similar brominated furanones have been reported, the latter being potent antibiofilm agents whose mechanism of action has been attributed to their capability to inhibit QS processes in bacteria [138]. Interestingly, a melanin pigment was informed with QQ activity. Table 7 shows these compounds’ families.

**Figure 7:**
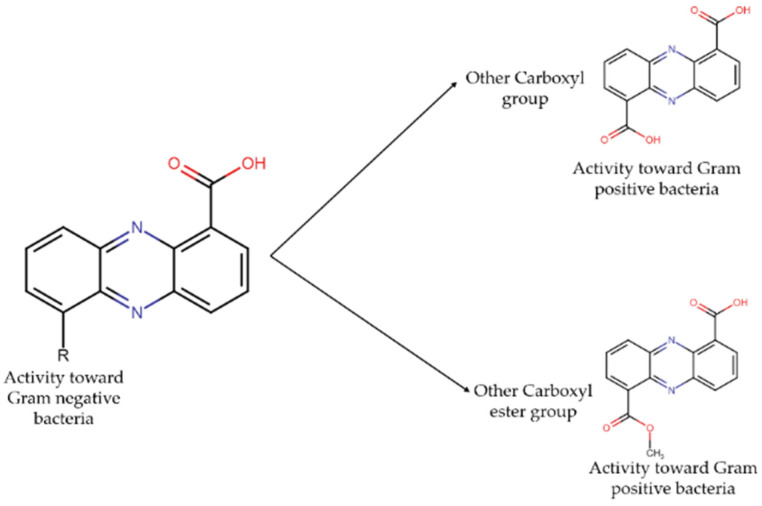
SAR of phenazine compound, modified from [27].

Some of the compounds reported with biological activities such as antibacterial, antimicrobial, antibiofilm, and QQ effects have been extensively studied and their structure–activity relationships (SAR) have been described; some of them are the following:

Phenazines, which are compounds with both antibacterial and antibiofilm activity, which is related to the presence of carboxylic acids on both C1 and C6 of the phenazine ring system, decreased the antibiofilm effect towards Gram-negative strains, but made these derivatives active against Gram-positive ones, particularly, *S. aureus.* Regarding that antibacterial activity, an analogous situation occurs in which the addition of another carboxylic acid or carboxyl ester at C-6 significantly decreased the inhibitory activity against Gram-negative bacteria and converts these phenazine derivates to be active against Gram-positive strains [27], as shown in Figure 7.

In the case of chlorinated bis-indole alkaloids, the SAR of these compounds, which showed antibacterial activity, reveals that the chlorine atom at C-6″ could be pivotal for conferring their bioactivity, thus providing hints on chemical modifications on bis-indole alkaloid scaffold in drug design [45].

Also, niphimycin is a type of macrolide with antibacterial activity against methicillin-resistant *S. epidermidis* (MRSE) and *S. aureus* (MRSA) [167]. Another type of macrolide is glycosidic antibiotics: similar to other macrolides, these compounds have antibacterial activity against Gram-positive organisms and are inactive against Gram-negative bacteria. This compound activity is related to the presence of hemiketal groups at C-11 and C-11’ in the structure. This is concluded because compounds that did not have this group showed an approximately two-fold decrease in activity against most strains [52]. Likewise, borrelidins J and K are macrolides that showed activity against MRSA, and their activity could enhance the cleavage of the ester bond. The cleavage of the ester bond in borrelidins makes them long-chain unsaturated fatty acids and it has been reported by previous studies that long-chain unsaturated fatty acids could exhibit strong activity against *S. aureus* by inhibiting the enoyl–acyl carrier protein reductase (FabI), which is the essential component in bacterial fatty acid synthesis [28].

Nocardiopsistins are angucycline compounds that belong to the polyketides family. These compounds presented antibacterial activity toward MRSA, and their activity is related to the presence of a hydroxyl group (-OH) at C3 in this structure [102].

Napyradiomycin is a large class of unique meroterpenoids with different halogenation patterns that present significant growth-inhibitory activity against MRSA. The specific mechanism of action for this family of meroterpenoids is not clear, however, studies about its SAR have shown that structural variations among the napyradiomycin metabolites, such as the different halogenation patterns or the presence or absence of the methyl group at C-7 among others, can attenuate or enhance their biological activities [113].

Lobophorin analogs are spirotetronate antibiotics with antibacterial activity against Gram-positive bacteria such as *Bacillus subtilis* and *S. aureus.* Their activity was related to compounds such as Lobophorin B, F H, I, and Lobophorin L, which has been related to the increase of the number of monosaccharide units in its structure, increasing inhibitory activity and indicating that monosaccharides might play a significant role in the antimicrobial activity of lobophorins [62,115]. In the same way, the antimicrobial activity showed by Lobophorins E and F is related to the absence of the hydroxyl group in C-32, which seems to enhance the bioactivity at a 416-fold improvement. On the contrary, the presence of the terminal sugar moiety is disadvantageous for the antimicrobial property [59].

Another compound that has reported SAR is Citreamicin, which is a xanthone commonly found in plants. It showed antibacterial activity against *S. aureus*; this may be due to the five-member nitrogen heterocycle in their structure. This five-member nitrogen heterocycle is similar to that in oxazolidinones, which are an approved class of antibiotics [84].

### 2.9. Biosynthetic Gene Clusters, BGCs

The capability of actinobacterial strains to produce bioactive secondary metabolites is considered to rely on their genomic potential, which typically contains many biosynthetic gene clusters (BGCs), including genes encoding for polyketide synthases (PKS) and non-ribosomal peptide synthetases (NRPS) [168]. However, nowadays, other biosynthetic gene clusters have been found, especially in marine actinobacteria, which, due to environmental conditions, are targets for the search for compounds with anti-infective activity that could provide alternative treatments for acne vulgaris. In addition to the PKS/NRPS clusters, in this study, other biosynthetic gene clusters have been reported such as the phenazine cluster, (this has been related to QQ and antibiofilm activity), which is directly involved in the production of phenazine compounds, the DSA cluster, related to the production of desotamides, the nes gene cluster, involved in the production of nenestatin A (Benzofluorene angucyclines), the abo cluster related to the aborycin compound, among others. Table 8 presents the details of the biosynthetic cluster genes reported in this study [168].

Natural products derived from these biosynthetic pathways have been extensively described for cultured and uncultured marine strains. Metabolites derived from marine actinobacteria include, among others, the polyketide synthase-derived abyssomicin C, a unique polycyclic polyketide from a marine *Verrucosispora* [97,130], salinisporamide A, from *Salinispora tropica* [108] that is currently in clinical trials as one of the most potent anticancer agents isolated until today [173], all isolated from the phylum of Actinomycetales.

**Figure 8:**
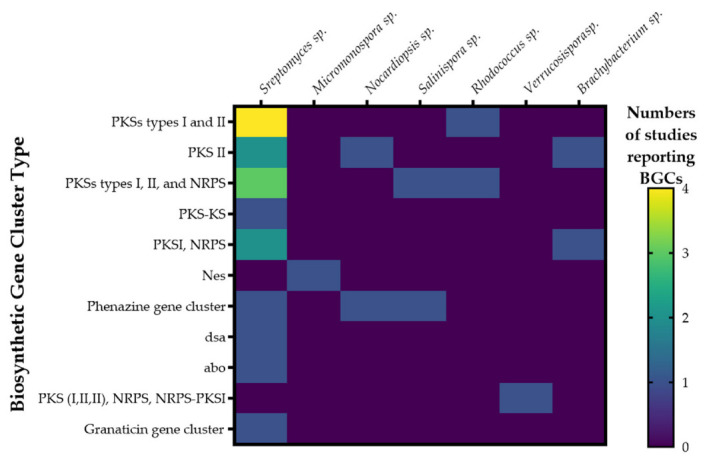
Heatmap of the number and type of biosynthetic gene clusters (BGCs) in the genomes of bioactive strains belongs to different genera collected in this study. Clusters are arranged top to bottom, beginning with the greatest number of BGC types in the top left. Strains are shown left to right by the highest number of BGCs. The most abundant BGCs were Type I and II PKSs followed by NRPS clusters for the *Streptomyces* genus. The color bar represents the number of studies that reported a type of BGCs, Purple to blue (minor values), blue to green (middle values), and green to yellow (high values).

BGCs sequences have been reported in marine actinobacteria isolated from a wide variety of environments and with a high occurrence variability. Of the articles included in this systematic review, only 21 reported the presence of biosynthetic gene clusters related to the biological activity of the promising strains. Of these, five articles reported the complete genomes and four reported the BGC sequences. Among the BGCs, the most common were type I and type II polyketide synthases (PKS-I, PKS-II), and nonribosomal synthetase (NRPS), mostly identified in *Streptomyces* sp., followed by *Salinispora* sp. isolated from marine sediments, as well as *Nocardipsis* sp., isolated from a sponge. This type of BGS has been the most studied; nevertheless, other BGCs have been reported in *Streptomyces* sp. such as the *abo* cluster, which is related to the synthesis of a compound with anti-infective activity, aborycin; the *dsa* cluster that is directly involved in the biosynthesis of the antibacterial compound desotamide, which has activity against *S. aureus* ATCC 29213, and methicillin-resistant *S. epidermidis* (MRSE) shhs-E1; phenazine cluster (*phe*), which has also been described in the genera *Nocardipsis* and *Salinispora*. Likewise, other BGCs have been found in genera such as Micromonospora, such as the *nes* cluster, involved in the biosynthesis of homo-dehydrorabelomycin E, which had antibacterial activity against *S. aureus* ATCC 29213, as presented in Figure 8. Despite this fact, it is important to note that the detection of genes associated with these biosynthetic clusters does not guarantee the expression of the genes involved in the production of secondary metabolites; notwithstanding, the detection of secondary metabolite biosynthetic pathways can be used as an indicator of metabolic potential, and suitable culture conditions are generally needed to express most of these pathways as well as the use of the appropriate targets to reveal the biological activity of the compounds [108].

## 3. Discussion

Microbial secondary metabolites are prevalent sources of natural products and they have been known as immense reservoirs of chemical classes of compounds with strong biological activities such as promising therapeutic potential [37].

Among the microorganisms, the actinobacteria phylum is one of the most known groups, being biologically active secondary metabolite producers, and it continues to represent an exciting source for the identification of novel natural products; due to this, it is considered the most economical and biotechnological important prokaryote source [101].

Out of these Actinobacteria, *Streptomyces* is the genera known as the most prolific, with many natural products with antibacterial, antifungal, antioxidant, antitumor activity, etc., from which products have been developed with a wide range of pharmaceutical applications contributing to a high number of antibiotics with current pharmaceutical applications, potentially useful to treat acne vulgaris [101]. Nevertheless, in the last years, other actinomycetes genera have received more attention as producers of commercially important secondary metabolites due to the probability of the rediscovery of novel compounds with new chemical structures from *Streptomyces* being increased [101], especially if they are obtained from terrestrial environments. Whereby, environments less explored as oceans, which cover about two-thirds of the Earth’s surface, have become important because they are considered a source in which microorganisms are submitted to extreme conditions and they are more challenging to culture compared to their terrestrial relatives. Therefore, the sea offers an enormous resource for novel compounds. The field of marine drug discovery has been growing over the past 20 years, with currently almost 35,000 research articles on natural products of marine origin [22].

The present review showed a significant increase of studies from 2002 to 2022, which demonstrates the interest in the marine environment to search for new bioactive compounds in addition to the need for the discovery of new compounds with anti-infective activity, finding that the majority of molecules reported are derived from *Streptomyces*, with a rising potential of finding new active compounds from rare actinobacteria genera such as *Nocardiopsis,* producing compounds with antibacterial, antimicrobial, anti-biofilm and QQ activity [29,137].

As expected, the antibacterial activity is the most reported biological activity and with the higher number of molecules discovered. These have very varied modes of action, such as affecting the membrane of the target bacteria and interrupting protein synthesis, among others. Likewise, in this systematic review, molecules, extracts, and fractions were reported as being highly active with MICs ranging from 0.01 to >1000 µg/mL. This shows that reported MICs are variable and that there is no consensus on the minimum value of the MIC to consider whether the compounds, fractions, or extracts are active and whether they have true pharmaceutical potential to produce commercial alternative treatments for acne vulgaris.

In addition, although there is a wide variety in the MICs reported, compounds with extremely low MICs are ideal, as this would allow the use of the compound in low proportions, this being more favorable than compounds that require a large amount to achieve the desired activity.

Likewise, some specific isolation sources have been prevalent, such as the marine sediment being the most frequent, becoming a reference hotspot for the bioprospecting of marine actinobacteria with antibiotic activities in the last decades [19]. The sea floor has been reported as a unique system with many forms of actinomycetes [174] and this is attributable to marine sediments, which are mixtures of complex organic and inorganic particles that have accumulated due to the accretion and erosion of the continents, oceanic biological activities, volcanic eruptions, and chemical processes within the ocean. Given their vast coverage, marine sediments harbor remarkably diverse microbial communities accounting for 12–45% of the total microbial biomass [23]. Proof of this is the fact that in compounds with antibacterial activity, the most predominant isolation source was marine sediment, followed by sponges, and ascidians, which are sessile marine invertebrates, making them vulnerable to predation and therefore are hypothesized to use host-associated bacteria that produce biologically active secondary metabolites for chemical defense [25].

Moreover, compounds with antibiofilm activity and metabolites with antibacterial and antibiofilm activity also have been isolated from sponges. It is well known that the sponges are of great biotechnological interest because these are well known for hosting a complex microbial consortium with the potential of producing biologically active secondary metabolites. Three-fourths of all discovered new bioactive microbial products from the oceans have originated from bacteria associated with marine invertebrates [175]. Two articles included in this systematic review with antibacterial, antibiofilm, and antimicrobial activity were reported by Joseph et al. and Sing et al., respectively, in which these bioactive compounds were isolated from a marine sponge symbiont, *Streptomyces pharmamarensis,* and marine-sponge-derived *Salinispora* sp., showing the enormous potential of marine sponge-associated actinomycetes that represent an exciting resource for the identification of new and novel natural products [110,134]. In the same way, another paper was reported by Hifnawy et al., in which two rare actinomycetes (*Micromonospora* sp. UR56 and *Actinokineospora* sp. EG49) were co-cultures and this led to the isolation of antibacterial metabolites of the phenazine class with antibiofilm, and cytotoxic properties [27].

Similarly, some compounds isolated from marine sponges, including angucyclines, antibacterial metabolites generating cell wall disruption in MRSA, have been reported previously [10,132]. Furthermore, one of the bacteria of interest in this paper is *S. epidermidis*, however, there are few articles reporting the action of actinobacterial compounds against this bacterium. Nevertheless, one article reported its growth and biofilm inhibition by *Streptomyces* sp. SBT348 extract [133] isolated from the marine sponge *Petrosia ficiformis*.

Concerning compounds with QQ activity, the sources from which the bacteria that produce them have been isolated are very varied: these are the intestines of marine fish, marine sediments, sponges, and water [29,137,138,140,141]. This may be due to the few studies that have so far been reported or have had their activity evaluated in extracts or isolated compounds of marine actinobacteria, indicating that there is no specific marine source for the isolation of marine actinobacteria with such activity.

Regarding places of isolation, two sites where more actinobacterial strains with anti-infective activity were isolated were the South China Sea and the Bay of Bengal in India. The former has emerged as a potentially abundant source of new species or genera of marine actinomycetes. Some new bioactive compounds, lobophorins E and F, were reported from marine actinomycetes isolated from the South China Sea [59]. The second is a well-known potential source for marine-derived bacteria rich in bioactive compounds [148] and is a point of access for diverse sets of marine fauna and flora, in particular sponges, sea anemones, sea cucumbers, sea urchins, soft corals, and many marine algae that, due to being little explored, have given rise to their bioprospecting as reported by Gandhimathi et al. [176].

The compounds most commonly produced by marine actinobacteria that have been recovered in this study are compounds with antibacterial activity against *S. aureus* and methicillin-resistant *S. aureus* (MRSA) (that could be present in skin diseases, but are also related), which cause a wide range of infections such as furuncles, pneumonia, osteomyelitis, endocarditis, bacteremia, etc. [171]. These same compounds in some cases have shown antibiotic activity against other Gram-positive bacteria such as *S. epidermidis*, *Bacillus* sp., vancomycin-resistant *Enterococcus faecalis* (VRE), among others, and to a lesser extent, against Gram-negative bacteria such as *E. coli*, *P. aeruginosa*, among others [133]. This phenomenon may be due to the morphological differences between Gram-positive and Gram-negative microorganisms. Whilst Gram-negative bacteria have an outer lipopolysaccharide membrane that makes the cell wall impermeable to lipophilic solutes, Gram-positive bacteria are more susceptible as they have a more permeable outer peptidoglycan layer [30]. However, this demonstrated the potential of compounds from marine actinobacteria to contribute to infectious disease control. This indicates a great possibility of using these compounds to treat acne vulgaris and the bacteria commonly associated with it, which are mainly Gram-positive bacteria.

In this same sense, it is noteworthy that few studies with activity against *S. epidermidis* [83] were retrieved, and there are none with activity against *Propionibacterium acnes*, currently renamed *C. acnes*, which is also an actinobacterium, but to date, there is no study on the action of compounds isolated from marine actinobacteria against this bacterium, which can become pathogenic due to unknown effects and participate in the development of the pathology of acne vulgaris. The fact that *C. acnes* is an anaerobe could increase the technical requirements to carry out the antibacterial activity screening; nevertheless, it is highly expected that the antibacterial compounds here described also have antibacterial activity against this bacterium.

It is important to point out that two of the antibiotics most currently used to treat acne were obtained from actinobacteria of the *Streptomyces* genus from soil samples. These compounds are erythromycin, belonging to the macrolide class, which has also been isolated from marine actinobacteria as reported in this systematic review, and clindamycin, a semi-synthetic derivative of lincosamide with a mechanism of action similar to macrolides, which binds to the 50S ribosomal subunit of bacteria, inhibiting protein synthesis [164]. This demonstrates the potential of actinobacteria as a source of new compounds for the treatment of acne vulgaris and the opportunity to study, search and develop compounds with antibacterial activity or QQ activity against this bacterium, the latter activity being a therapeutic target since in most cases it does not affect bacterial growth, which would be positive for *C. acnes* since it is a bacterium that, in a normal environment of the skin, protects from the invasion of pathogenic bacteria, contributing to its homeostasis.

Regarding antibacterial metabolites, various studies have reported bioactive metabolites that belong to the polyketide family, it being one the most isolated families from marine actinobacteria. Among these were found aromatic polyketides as described by Dong et al., Ahamad et al., and Govindarajan et al. [61,68,151], angucyclines described by Akhter et al. [54], polyketide–terpenoid as Naphthoquinone, reported by Shen et al. [37], macrolides, described by Braña, Zhang and Wu [52,67,107,152], etc. Likewise, some chlorinated compounds were frequent, such as chlorinated bis-indole alkaloids and chlorinated 3-phenylpropanoic acid described by Song et al. [45] and Shaala et al. [124]; this may be due to the concentrations of chloride and bromide ions in the ocean [177]. Interestingly, marine-derived bis-indole compounds typically contain halogen atoms in their structures. Such halogenated bis-indole alkaloids display potent cytotoxic or antibacterial activities or both, and they are thus considered promising anti-cancer or antibacterial leads. In the same way, a series of marine-derived chlorinated bis-indoles were shown to inhibit methicillin-resistant *S. aureus* (MRSA) pyruvate kinase significantly, with their halogenated indole ring being implicated as a critical pharmacophore [45]; this has been reported by Wang et al. [177] and is well known that marine actinomycetes produce a variety of halogenated compounds with diverse structures and a range of biological activities owing to their unique metabolic pathways [31,177].

Similarly, compounds in the bis-indole family are ubiquitously distributed in plants and microorganisms, similar to phenolic compounds, which can be defined as plant substances, are the most widely distributed in the plant kingdom, and are the most abundant secondary metabolites of plants [178]. However, some of them have been isolated from marine actinobacteria as described by Siddharth and Rai [101], specifically, from rare actinomycetes *Nocardiopsis* sp. This metabolite (4-bromophenol, a bromophenol derivative) exhibited a significant antioxidant activity through DPPH and ABTS assays, as was expected due to the antioxidant capacity that has been described in these compounds; in addition, it showed broad-spectrum inhibitory activity against MRSA, *Klebsiella pneumonia* ATCC 13883, *B. subtilis* ATCC 6633 [101]. Likewise, other plant-derived compounds have been isolated from marine actinobacteria as Cinnamaldehyde, produced by *Streptomyces chartreusis*, which showed antibacterial activity, and other studies reported its effect on the swarming motility of *P. aeruginosa*, which is related to quorum sensing in this bacterium, which shows the possible ability of Cinnamaldehyde to inhibit quorum sensing [174].

As for the compounds, there is a wide diversity, finding polyketides, macrolides, quinolones, terpenes, phenazines, naphthoquinones, and phenolic compounds that displayed antibacterial, antimicrobial, antibiofilm activity, and QQ; within these are some compounds that mainly have been discovered in plants, but nowadays have been discovered in marine actinobacteria, such as cinnamaldehyde, flavonoids, and xanthone natural products, which exhibit a wide array of bioactivities including antioxidant, antibacterial, antimalarial, antituberculosis and cytotoxic activities as reported earlier [179].

Most of the compounds obtained from actinobacteria have been isolated using organic solvents. Among the articles collected in this systematic review, the most reported was ethyl acetate, which has a medium to high polarity. This solvent is described as the ideal solvent for obtaining metabolites with antibacterial activity. This may be because it is possible that actinobacteria, especially streptomyces, produce semipolar antibacterial compounds so that they can be extracted by solvents with the same polarity as ethyl acetate, as mentioned in the study by Kurnianto et al. [180]. Likewise, Satish et al. evaluated the activity of extracts obtained from different solvents such as chloroform, butanol, and ethyl acetate against MRSA, finding that only the extracts obtained with the latter exhibited antibacterial activity [87].

On the other hand, traditionally, marine invertebrates are considered a prolific source of exceptional natural products, with a diverse range of biological activities. However, current studies on invertebrate-associated microbial communities are revealing microorganisms as the real producers of many of these compounds. In this study, one article with *Streptomyces* strains was reported with QQ and antimicrobial activity isolated from the gut of marine fish *Rastrelliger kanagurta* [141]. This compound was not identified, however, in this study the findings revealed that there is a wide variety of compounds of the family, with polyketides being the most frequent, as expected, because they have been the most studied and are synthesized by the enzyme polyketide synthase, encoded by PKS genes against which genetic mobilization through horizontal gene transfer (HGT) has been reported with a high frequency, and this could be due to multiple factors. Some PKSs are encoded on plasmids or located within pathogenic islands, which facilitate gene transfer through conjugation, transposition, or transduction, as was reported by Nivina et al. [181].

In this same sense, the PKS gene cluster was the most reported, together with NRPS and the *phe* gene cluster, however, the detection of genes associated with these biosynthetic clusters does not guarantee the expression of genes involved in the production of secondary metabolites due to recent studies have demonstrated that the abundance of biosynthesis gene clusters in actinobacteria genomes do not appear to be expressed under standard laboratory culture conditions. Activation of these gene clusters would considerably enhance the ability to discover novel natural products. Studies by Xu et al. have shown that LaCl3 induced antifungal or antibacterial activities in strains that did not show such activities under normal cultivation conditions [167]. In addition, the culture condition such as agitation speed, temperature, pH, etc., apart from helping improve the performance of compounds, could also be related to the expression of the biosynthetic gene cluster. Furthermore, carbon and nitrogen sources have been reported with a profound influence on secondary metabolite production; regarding carbon sources, glucose favors a high growth rate, nevertheless, this represses secondary metabolite production through carbon catabolite repression [182,183]. Due to this, other sources have been used as starch; for this reason, glucose was reported in this review with less frequency compared to starch. Regarding the nitrogen source, ammonium is reported as the preferred nitrogen source for most actinobacteria; its presence in high concentrations is positively related to the growth rate, however, it delays the onset of secondary metabolite production. On the contrary, nitrate can be assimilated by actinobacteria as an alternative nitrogen source. Interestingly, the nitrate excess enhances secondary metabolite production in actinobacteria [182], which explains why nitrate has been reported in twice as many articles as ammonium in this systematic revision.

Respecting the antibiofilm activity, there are about 5027 anti-biofilm agents against Gram-positive and -negative bacteria, and fungi have been reported between 1988 and 2017 [133]. However, up to date, few have been successfully translated to the market for clinical and medical applications or against whom bacteria have developed action mechanisms, because of this is required to continue in the search for new options and despite the huge expectations on synthetic molecules with effective antimicrobial properties, natural products are still worthy of promise as reported by Newman and Cragg [30,184].

Although the compounds with QQ activity were few, the present investigation confirms the ability of actinobacteria to produce secondary metabolites with this effect, being one of the novel approaches to counter the drug-resistant bacteria and target therapeutic that could be inhibited the virulence factors of some bacteria such as *C. acnes* and generate new treatment options to acne vulgaris disease.

Seeing these results in an integrated manner, it is possible to guide research towards the isolation of marine actinobacteria obtained from sediments and marine invertebrates, paying more attention to the *Streptomyces* genus, and looking for families of compounds such as polyketides, macrolides, phenazines, among others. In the same way, the variation of the culture condition may promote the production of bioactive metabolites, especially carbon and nitrogen sources.

In short, our results reinforce the need to further explore marine actinomycetes and their enormous potential of them as a rich source of novel metabolites relevant for biotechnological applications.

## 4. Materials and Methods

### 4.1. Search Strategy

A systematic search was conducted in PubMed, Scopus, and Web of Science (WOS) without limits of timeframe (The first search was in May 2021 and the last updated in April 2022). The search strategy for all databases included the descriptors: “streptomyces”, “actino”, “acne”, “antibacterial”, “quorum quenching” and other terms combined with Boolean operators AND and OR and it defined as follows.

((streptomyces OR action *) AND (acne * OR “staphylococcus epidermidis” OR “staphylococcus aureus” OR “cutibacterium acnes” OR “propionibacterium acnes”) AND (antibacterial OR quorum OR “quorum quenching”)).

“Acne” was used instead of “acne vulgaris” as it is more general and commonly used and the term “quorum” was included for researchers that used quorum-sensing inhibitors instead of “Quorum Quenching”.

In addition, for the synthesis, the papers were grouped by the type of biological activity reported.

### 4.2. Eligibility Criteria

Studies were included in this systematic review to see if they met all the following eligibility criteria:

Original research articles, studies on extraction of compounds or extracts or metabolites derived from marine actinobacteria strains, and studies evaluating the activities of antibacterial, antimicrobial, anti-biofilm, and quorum quenching.

The following were considered to be exclusion criteria: compounds or extracts isolated from soil actinobacteria or another environment different from marine, compounds or extracts obtained from microorganisms other than actinobacteria, compounds were not identified, reviews, communication, and letters to the editor were not considered and articles whose language was not English.

Three researchers performed all the literature selection steps individually and then discussed the differences within the research team. An article was eligible to be included in the review when at least two authors indicated that it met the inclusion/exclusion criteria. Eligible articles were read at a full-text level and those who met the inclusion/exclusion criteria were selected to carry out the data extraction.

### 4.3. Data Extraction

Data were extracted and sorted by the title of studies, author, year, the number of strains, isolation country, isolation source (sediment, sponge, seawater, mangrove, coral, marine invertebrates, and so on), genus of actinobacteria (*Streptomyces* sp., *Nocardiopsis* sp., *Micromonospora* sp., *Verrucosispora* sp., *Salinispora* sp., among others), type of activity (antibacterial, antimicrobial, antibiofilm, quorum-sensing inhibition), extracts or compounds used, the organic solvent used to get the extracts or compounds (EtOAc, MeOH, Butanone, Butanol, Methanol, Acetone, Chloroform, Dichloromethane, or the combination of them), the family of compounds, genes associated with the compounds’ production, the biosynthetic gene clusters (BGCs) and the structure of the compounds if reported.

## 5. Conclusions

The marine ecosystems are one of the most dynamic, under-explored environments and are a natural reservoir of metabolites with a wide spectrum of biological activities. *Streptomyces* sp. remains the most prolific genus of actinobacteria in the phylum, however, the so-called rare actinobacteria have gained interest due to the variety of compounds they can produce, such as those that show antibiofilm activity and quorum quenching. In the same way, marine sediments and sponges were the most outstanding source to isolate bioactive actinobacteria. Regarding compounds with antibacterial activity, polyketides were most frequently comprised of angucyclines, aromatics polyketides, and naphthoquinones, among others, followed by phenazines which displayed antibacterial, antimicrobial, and quorum-sensing inhibition, finding an exciting potential in this type of secondary metabolite. Likewise, compounds originally found in plants were reported to be isolated from marine actinobacteria, evidence that the bioactivity of some plants or animals like fishes is due to microorganisms and not to the host organism. Furthermore, it was evident that there are few studies of the compounds obtained from marine actinobacteria with antibacterial, antibiofilm, or QQ activity against *C. acnes*, giving us a wonderful opportunity to investigate future studies in this interesting area. Finally, biosynthetic gene clusters in the production of secondary metabolites in actinobacteria play an important role, and although the presence of this in the genome of actinobacteria does not imply that they will be expressed, they are indicators of the potential of strains to produce compounds and it was clear that in most cases that they must be activated through some strategies such as co-culture, stress-generated external factors such as pH, temperature, agitation speed, variation of co-culture conditions and so on. This makes evident the need to sequence the genomes, since these allow us to know the bacteria in-depth and put into practice different strategies, establish the relationship between gene clusters of genes and functions, postulating this methodology as an alternative for the extraction of metabolites, its performance and use. In short, the findings in this research support the evidence of the potential of marine-derived actinobacteria to produce anti-infective compounds and suggested the search for this microorganism of compounds with novel approaches as QQ.

## Figures and Tables

**Table 2:** Antimicrobial activity of actinobacterial crude extracts or compounds.

Genus	Pathogen Target	Compounds/Extracts	MIC (μg/mL)	Ref.
*Streptomyces* sp.	*S. aureus* FDA209P JC-1	Chlorinated α-lapachone	12.5	[31]
*Streptomyces* sp.	MRSA ^1^	Streptoindoles A	25	[32]
		Streptoindoles B	7	[32]
		Streptoindoles D	25	[32]
*Streptomyces* sp.	MRSA ^1^	Streptoglutarimides A−J	9–11	[111]
*Streptomyces* sp.	*S. aureus*	Nitricquinomycin C	17	[112]
*Streptomyces* sp.	MRSA ^1^	Napyradiomycin D1	12–24	[113]
*Streptomyces* sp.	*S. aureus* ATCC 33591	Polyketide antibiotic SBR-22	64	[114]
*Streptomyces* sp.	*S. aureus* ATCC 29213	Lobophorins F	6.25	[115]
*Streptomyces* sp.	*S. aureus*	Polyketide related antibiotic	37.5	[30]
*Streptomyces* sp.	MRSA ^1^	Actinomycin D	0.08	[116]
		Actinomycin V	0.08	[116]
		Actinomycin X_0_β	0.61	[116]
*Streptomyces* sp.	MRSA ^1^	Niphimycins C	4–32	[117]
	MRSE ^2^	Niphimycin Iα	4–32	[117]
*Streptomyces* sp.	*S. aureus* ATCC 25923	Trihydroxylflavanone ^3^	32	[118]
		Tetrahydroxylchalcone ^4^	1	[118]
*Streptomyces* sp.	*S. aureus*	Anthracycline analogues	20	[119]
		β-rhodomycin-II	40	[119]
*Streptomyces* sp.	*S. aureus*	DMBPO ^5^	>1000	[120]
*Streptomyces* sp.	*S. aureus* ATCC 25923	Chromomycin A9	0.03	[121]
		Chromomycin Ap	0.13	[121]
		Chromomycin A2	0.06	[121]
		Chromomycin A3	0.13	[121]
*Streptomyces* sp.	MRSA ^1^	Streptopyrazinones A–D	58–65	[122]
		N-acetyl-L-isoleucine-L-leucinamide	65	[122]
*Streptomyces* sp.	MRSA ^1^	4-dehydro-4a-dechloronapyradiomycin A1	4–8	[123]
		Napyradiomycin A1	0.5–1	[123]
*Streptomyces* sp.	*S. aureus*	3-propanoic acid ^6^	32	[124]
		Propanoic acid methyl ester ^7^	64	[124]
		3-(3-chloro-4-hydroxyphenyl) propanoic acid	32	[124]
*Streptomyces* sp.	*S. aureus* (ATCC 6538)	Natural cyclic peptide	1.25	[125]
	MRSA ^1^		12.5	[125]
	*S. aureus* (ATCC 6538)	Cyclic peptides	0.025–0.156	[125]
	MRSA ^1^	Cyclic peptides	0.1–0.78	[125]
*Streptomyces* sp.	*S. aureus*	Extracts A758	6.25	[126]
		Extracts A759	500	[126]
		Extracts A760	100	[126]
		Extracts A765	3.125	[126]
*Streptomyces* sp.	MRSA ^1^	Novobiocin	0.25	[127]
		Desmethylnovobiocin	16	[127]
		5-Hydroxynovobiocin	8	[127]
*Kocuria marina*	*S. aureus*	Kocumarin	10	[128]
	MRSA ^1^	Kocumarin	10	[128]
*Rhodococcus* sp.	*S. aureus*	n-butanol	9.3	[34]
		fraction		
		EtOAc fraction	12.6	[34]
*Marinispora* sp.	MRSA ^1^	Marinomycin A	0.130	[129]
		Marinomycin B–C	0.49	[129]
		Marinomycin D	2.43	[129]
*Verrucosispora* sp.	*S. aureus*	(2-(hydroxymethyl)-3-(2-(hydroxymethyl)-3-methylaziridin-1-yl) (2-hydroxyphenyl) methanone	3.4	[130]

^1^ MRSA: Methicillin-resistant *Staphylococcus aureus*. ^2^ MRSE: Methicillin-resistant *Staphylococcus epidermidis.* ^3^ Trihydroxylflavanone: lavandulyl-7-methoxy-5,20,40-trihydroxylflavanone. ^4^ Tetrahydroxylchal-cone 50-lavandulyl-40-methoxy-2,4,20,60-tetrahydroxylchalcone. ^5^ DMBPO: 5-(2,4-dimethylbenzyl) pyrrolidin-2-one Information no reported. ^6^ 3-propanoic acid: 3-(3,5-dichloro-4-hydroxyphenyl) propanoic acid. ^7^ Propanoic acid methyl ester: 3-(3,5-dichloro-4-hydroxyphenyl) propanoic acid methyl ester.

**Table 3:** Antibacterial and anti-biofilm activity of actinobacterial crude extracts or compounds from *Streptomyces* genus.

Genus	Pathogen Target	Compounds/Extracts	MIC (μg/mL)	Ref.
*Streptomyces* sp.	MRSA ^1^	Compound PVI331	1	[132]
*Streptomyces* sp.	MRSA ^1^	8-O-metyltetrangomycin	2	[10]
*Streptomyces* sp.	MRSE ^2^ RP62A	Compound (SKC3)	31.25	[133]
*Streptomyces* sp.	MRSA ^1^	PVI401	0.5	[134]
		PVI402	2	

^1^ Methicillin-resistant *Staphylococcus aureus*. ^2^ Methicillin-resistant *Staphylococcus epidermidis*.

**Table 5:** Marine actinobacteria with Quorum Quenching (QQ) activity.

Source	Genus	Disrupter QS System	Biosensor Strains	Ref.
Gut of marine fishes	*Streptomyces* sp.	AI-1: AHL	*C. violaceum* and *Serratia marcescens.*	[141]
NA ^1^	*Streptomyces* sp.	AI-1: AHL, AI-2: LuxS	*C. violaceum CV026* and *Vibrio harveyi BB170*	[138]
Marine Sponge	*Streptomyces* sp.	AI-1: AHL: LasI	*Pseudomona*- Molecular docking.	[140]
Marine sediment	*Nocardiopsis* sp.	AI-1: AHL	*C. violaceum 12472*	[29]
Seawater	*Nocardiopsis* sp.	AI-1: AHL	*C. violaceum (MTCC 2656)*	[137]

^1^ Information no reported.

**Table 6:** Family compounds with antibacterial activity.

Compound	Frequency	Constituents	Ref.
Polyketide	19	Naphthoquinone-based meroterpenoids Naphthoquinone Derivatives	[37] [25]
		Chlorinated Meroterpenoids (Merochlorins G–J)	[78]
		Angucycline	[23,42,50,54,100,102]
		Aromatic Polyketides	[61,68,151]
		Polyketide ^1^	[72]
		Compound 1 ^2^	[43]
		Macrolides ^3^	[52,57,67,96,107,132,152]
Phenolic compound	1	Bromophenol derivative	[101]
Phthalate	1	Bis (2-ethylhexyl)	[101]
Acetamide	2	4-methoxyacetanilide	[18]
Alkaloids	3	2-ethylhexyl 1H-imidazole-4-carboxylate	[97]
		butyl 1Himidazole-4-carboxylate	[97]
		Chlorinated bis-indole alkaloids	[45]
		Indolizinium alkaloid	[58]
Pyrrole	3	Chlorinated Bisindole Pyrrole	[106]
		Pyrrole-derivative	[41,60]
Chromopeptides	6	Actinomycins (X_0β_, X_2_, D, D1–D4, A)	[56]
		Neo-actinomycin A, B, actinomycins D and C4, X_2_,)	[64,77,80,153]
Cyclo peptides	3	Desotamides A–D	[154]
		cyclo(l-Val-l-Pro),	[79]
		cyclo-(l-Pro-4-OH-l-Leu)	[55]
Antracycline	1	Bisanhydroaklavi-none 1-Hydroxybisanhydroaklavinone	[19]
Marinopyrroles	1	(−)-marinopyrroles A	[70]
		(−)-marinopyrroles B	
Phenazines	5	phenazine-1,6-dicarboxylate, phencomycin, tubermycin	[27] [63]
		Streptophenazines G	
		1,6-Dihydroxy phenazine, dimethoxy phenazine Actinomycins D1 and D2	[155] [41]
Spirotetronate antibiotics	2	Lobophorins L and M	[62]
		Lobophorins E	
Proteins	2	Enzyme PA720 (Thermophilic Hemoglobin-degrading Protease)	[156]
		β-lactamase inhibitory protein	[157]
Pyranonaphthoquinones	3	Medermycin-type naphthoquinones	[158]
		Medermycin derivative	[51]
		Lactoquinomycin A (LQM-A)	[53]
Quinomycin family antibiotics	1	Quinomycin G	[55]
Quinona	1	1- hydroxy-1-norresistomycin	[38]
Siderophore native	3	S1, S2, S3 ^4^	[57,144,159]
Thiazolyl Peptide Antibiotic Family	1	Kocurin	[108]
Pigment	1	Melanin pigment	[137]
Aminofuran natural products	1	Proximicin F and G	[94]
Type I lasso peptide natural products	1	Aborycin	[48]
Natural product class diazaanthraquinone	1	Diazaanthraquinone	[160]
Benzoic acid	1	2,4-dichloro-5-sulfamoyl benzoic acid	[44]
4-oxazolidinone antibiotics	1	Lipoxazolidinone A, B and C.	[105]
Cyslabdan-like compound	1	Cyslabdan-like compound	[93]
Benzene Derivative	1	1,3-Benzodioxole	[81]
Flavonoids	3	Citreamicin *θ* A	[84]
		Citreamicin *θ* B	
		Citreaglycon A	
		Dehydrocitreaglycon A	

^1^ Polyketide: Compound name no identified. ^2^ Compound 1: [2-hydroxy-5-((6-hydroxy-4-oxo-4Hpyran-2-yl) methyl)-2-propylchroman-4-one]. ^3^ Polyketide: Elaiophylin Derivatives, Nargeninas, Desertomycin G, Kendomycin analogues, N-Arylpyrazinone Derivative. ^4^ S1: 5,6-dihydro-1,8-dihydroxy-3-methylbenz[a]anthracene-7,12-quinone; S2: 1,4-dihidroxy-2-(3-hydroxybutyl)-9, 10-antraquinone; S3: Desferrioxamine B and the New Desferrioxamine B2.

**Table 7:** Family compounds with QQ activity.

Compound	Frequency	Constituents	Ref.
Fatty acyl compounds	1	13Z-Octadecenal.	[140]
Phenoxazines	1	Questiomycin A	[29]
		2-hydroxyacetate-3-hydroxyacetamido-phenoxazine (HHP)	[29]
Lactones	1	Butenolide	[138]
Pigment	1	Melanin	[137]
Strain IM20 ^1^	1	NA ^2^	[141]

^1^ Compound not identified. ^2^ Information not reported.

**Table 8:** Biosynthetic gene clusters identified in marine actinobacteria reported in this study.

Genus	BGS	Genes	Metabolites Production	Ref.
*Streptomyces* sp.	PKS gene cluster	PKS-I and PKS-II Genes	Polyketide	[20,30]
			Angucycline	[23]
		PKS-II Genes	Angucyclinone derivatives	[146]
		PKS-KS	NA ^1^	[169]
		PKS	Niphimycins	[117]
	PKS/NRPS	PKS II	Analogue of paulomenol	[103]
		NA ^1^	Antimycin A analogues	[77]
		NA ^1^	NA ^1^	[168,170]
		NRPS, PKS Type I, II, and III	Naphthoquinone antibiotics	[25]
	NRPS gene cluster	NRPS-A	NA ^1^	[169]
		NA ^1^	NA ^1^	[49]
	Aborycin biosynthetic gene cluster (abo)	NA ^1^	Aborycin	[48]
	Lassopeptide cluster	NA ^1^	Lasso peptide family	[48]
	Phenazine cluster	phzE and phzF	Streptophenazines (Phenazines)	[83]
	dsa cluster	DsaA y DsaN, dsaB y dsaJ	Desotamides	[154]
	PKS/terpenoid biosynthetic pathways	NA ^1^	Napyradiomycin derivatives (Terpenoids)	[71,113,171]
*Micromonospora* sp.	nes gene cluster	NA ^1^	nenestatin A (Benzofluorene angucyclines)	[100]
Co-culture of *Actinokineospora* sp. and *Micromonospora* sp.	NA	NA ^1^	Phenazine	[27]
*Nocardiopsis* sp.	PKS/NRPS	NA ^1^	Polyketide	[170]
	PKS gene cluster	PKS-II	α-pyrone compound	[103]
		ACP synthase α-subunit (KSα), β-subunit (KSβ) and acyl carrier protein (ACP)	Angucyclines	[102]
		PKS-II	Angucycline	[102]
		phzE	Phenazines	[155]
*Rhodococcus* sp.	NRPS/NRPS	NA ^1^	NA ^1^	[22]
	PKS/NRPS	NA ^1^	Polyketide	[170]
*Salinispora* sp.	PKS gene cluster	PKS I, II	Rifamycin B	[103]
	PKS/NRPS	NA ^1^	Polyketide	[170]
*Verrucosispora* sp.	PKS gene cluster	PKSI (pks1 and pks2), two PKSII (pks3 and pks4), PKSIII (pks5);	New salicylic derivative, brevianamide F, abyssomicin B	[95]
	NRPS gene cluster	NA ^1^		
	Terpene clusters	terp1, terp2, terp3 and terp4		
	NRPS-PKSI hybrid clusters	np1 and np2		
	Lanthipeptide clusters	lant1 and lant2		
	Siderophore cluster	sid		
*Brachybacterium paraconglomeratum*	NRPS/PKS	NRPS genes, PKS type I genes, and PKS type II gene	NA ^1^	[172]

^1^ Information no reported.

## Data Availability

Data supporting reported results can be found in this document and the Supplementary Materials. If they become required, please request them by mail at luisa.villamil@unisabana.edu.co.

## Supplementary Materials

The following supporting information can be downloaded at: https://www.mdpi.com/article/10.3390/antibiotics11070965/s1, Table S1: PRISMA checklist, Table S2: Source of isolation and type of activity of actinobacterial strains reported in this systematic review. Table S3: Antibacterial activity of actinobacterial crude extracts or compounds presented through inhibition zone (mm). Table S4: Antimicrobial activity of actinobacterial crude extracts or compounds presented through inhibition zone (mm). References [185,186,187,188,189,190,191,192,193,194,195,196,197,198,199,200,201,202,203,204,205,206,207,208,209,210,211,212,213,214,215] are cited in the supplementary tables.
