# Supplementary material for: Marine Actinobacteria a New Source of Antibacterial Metabolites to Treat Acne Vulgaris Disease—A Systematic Literature Review
Source: Antibiotics (Basel). 2022 Jul 18;11(7):965. doi: 10.3390/antibiotics11070965 (PMC9311749; doi:10.3390/antibiotics11070965)
Supplement: Supplementary file 1 [file antibiotics-11-00965-s001.zip › Table S2.pdf]

Table S2. Source of isolation and type of activity of actinobacterial strains reported in this systematic review.

Marine Source	Bioactivity	No. Papers	No. Strains	Ref.
Sediment	Antibacterial	70	114	[1–10][11–20][21–30][31–40][41–50][51–60][61–70]
	Antimicrobial	25	50	[71–80][81–90] [91–95]
	Antibiofilm/QQ	2	4	[96,97]
	Antibacterial/Antiviral	2	19	[98,99]
Marine algae	Antibacterial	7	7	[100–106]
	Antimicrobial	2	2	[107,108]
Mangrove	Antibacterial	6	7	[109–114]
	Antibiofilm	1	1	[115]
Water	Antibacterial	4	6	[116–119]
	Antimicrobial	2	NA[1]	[120,121]
	Antibacterial/Anti-biofilm/QQ	1	2	[122]
Marine invertebrates	Antibacterial	20	38	[123–132] [133–142]
	Antimicrobial	19	79	[143–152] [153–161]
	Antibacterial/antibiofilm/QQ	1	1	[162]
	Antibacterial/antibiofilm	5	6	[163–167]

Seagrasses	Antibacterial	2	2	[168,169]
Fishes	Antibacterial	1	2	[170]
	Antimicrobial	1	1	[171]
NA[1]	Antibacterial	5	5	[172–176]
	Anti-biofilm/QQ	1	NA[1]	[177]
Total		177	305	

[1]Information no reported

References

1. Cheng, Y.-B.; Jensen, P.R.; Fenical, W. Cytotoxic and Antimicrobial Napyradiomycins from Two Marine-Derived, MAR 4 Streptomyces Strains. *European J. Org. Chem.* **2013**, *2013*.
2. Fang, C.; Zhang, Q.; Zhu, Y.; Zhang, L.; Zhang, W.; Ma, L.; Zhang, H.; Zhang, C. Proximicins F and G and Diproximicin A: Aminofurans from the Marine-Derived Verrucosispora Sp. SCSIO 40062 by Overexpression of PPtase Genes. *J. Nat. Prod.* **2020**, *83*, 1152–1156, doi:10.1021/acs.jnatprod.9b01185.
3. Hughes, C.C.; Prieto-Davo, A.; Jensen, P.R.; Fenical, W. The Marinopyrroles, Antibiotics of an Unprecedented Structure Class from a Marine Streptomyces Sp. *Org. Lett.* **2008**, *10*, 629–631.
4. Dholakiya, R.N.; Kumar, R.; Mishra, A.; Mody, K.H.; Jha, B. Antibacterial and Antioxidant Activities of Novel Actinobacteria Strain Isolated from Gulf of Khambhat, Gujarat. *Front. Microbiol.* **2017**, *8*, doi:10.3389/fmicb.2017.02420.
5. Siddharth, S.; Vittal, R.R. Isolation, Characterization, and Structural Elucidation of 4-Methoxyacetanilide from Marine Actinobacteria Streptomyces Sp. SCA29 and Evaluation of Its Enzyme Inhibitory, Antibacterial, and Cytotoxic Potential. *Arch. Microbiol.* **2019**, *201*, 737–746, doi:10.1007/s00203-019-01634-y.
6. Ballav, S.; Kerkar, S.; Thomas, S.; Augustine, N. Halophilic and Halotolerant Actinomycetes from a Marine Saltern of Goa, India Producing Anti-Bacterial Metabolites. *J. Biosci. Bioeng.* **2015**, *119*, 323–330, doi:10.1016/j.jbiosc.2014.08.017.
7. Priyanka, S.; Jayashree, M.; Shivani, R.; Anwesha, S.; Bhaskara Rao, K. V.; I, A.E. Characterisation and Identification of Antibacterial Compound from Marine Actinobacteria: In Vitro and in Silico Analysis. *J. Infect. Public Health* **2019**, *12*, 83–89, doi:10.1016/j.jiph.2018.09.005.
8. Shen X. Wang X., H.T.D.Z.L.S. Naphthoquinone-Based Meroterpenoids from Marine-Derived Streptomyces Sp. B9173. **2020**, *10*.
9. Kock I. Maskey R.P., B.M.A.F.H.E.L.H. 1-Hydroxy-1-Norresistomycin and Resistoflavin Methyl Ether: New Antibiotics from Marine-Derived Streptomycetes. **2005**, *58*.
10. Abd-Elnaby, H.; Abo-Elala, G.; Abdel-Raouf, U.; Abd-Elwahab, A.; Hamed, M. Antibacterial and Anticancer Activity of Marine Streptomyces Parvus: Optimization and Application. *Biotechnol. Biotechnol. Equip.* **2016**, *30*, 180–191, doi:10.1080/13102818.2015.1086280.

11. Iniyan, A.M.; Joseph, F.R.S.; Kannan, R.R.; Vincent, S.G.P. Anti-MRSA Potential of Phenolic Compound Isolated from a Marine Derived Actinomycete Micromonospora Sp. ICN36. *Indian J. Geo-Marine Sci.* **2016**, *45*, 1279–1287.
12. Ahmad, S.; Nazir, M.; Tousif, M.I.; Saleem, M.; Mustafa, R.; Khatoon, T. A New Polyketide Antibiotic from the Marine Bacterium Streptomyces Sp. PGC 32. *Chem. Nat. Compd.* **2019**, *55*, 1–4, doi:10.1007/s10600-019-02602-0.
13. Sabido, E.M.; Tenebro, C.P.; Suarez, A.F.L.; Ong, S.D.C.; Trono, D.J.V.L.; Amago, D.S.; Evangelista Jr., J.E.; Reynoso, A.M.Q.; Villalobos, I.G.M.; Alit, L.D.D.; et al. Marine Sediment-Derived Streptomyces Strain Produces Angucycline Antibiotics against Multidrug-Resistant Staphylococcus Aureus Harboring SCCmec Type 1 Gene. *J. Mar. Sci. Eng.* **2020**, *8*, doi:10.3390/jmse8100734.
14. Yi, W.; Li, Q.; Song, T.; Chen, L.; Li, X.-C.; Zhang, Z.; Lian, X.-Y. Isolation, Structure Elucidation, and Antibacterial Evaluation of the Metabolites Produced by the Marine-Sourced Streptomyces Sp. ZZ820. *Tetrahedron* **2019**, *75*, 1186–1193, doi:10.1016/j.tet.2019.01.025.
15. Cheng, C.; Macintyre, L.; Abdelmohsen, U.R.; Horn, H.; Polymenakou, P.N.; Edrada-Ebel, R.; Hentschel, U. Biodiversity, Anti-Trypanosomal Activity Screening, and Metabolomic Profiling of Actinomycetes Isolated from Mediterranean Sponges. *PLoS One* **2015**, doi:10.1371/journal.pone.0138528.
16. Kurata, A.; Sugiura, M.; Kokoda, K.; Tsujimoto, H.; Numata, T.; Kato, C.; Nakasone, K.; Kishimoto, N. Taxonomy of Actinomycetes in the Deep-Sea Calyptogena Communities and Characterization of the Antibacterial Compound Produced by Actinomadura Sp DS-MS-114. *Biotechnol. Biotechnol. Equip.* **2017**, *31*, 1000–1006, doi:10.1080/13102818.2017.1342563.
17. McArthur, K.A.; Mitchell, S.S.; Tsueng, G.; Rheingold, A.; White, D.J.; Grodberg, J.; Lam, K.S.; Potts, B.C.M. Lynamicins A-E, Chlorinated Bisindole Pyrrole Antibiotics from a Novel Marine Actinomycete. *J. Nat. Prod.* **2008**, *71*, 1732–1737, doi:10.1021/np800286d.
18. Yang, L.; Hou, L.; Li, H.; Li, W. Antibiotic Angucycline Derivatives from the Deepsea-Derived Streptomyces Lusitanus. *Nat. Prod. Res.* **2020**, *34*, 3444–3450, doi:10.1080/14786419.2019.1577835.
19. El-Naggar M.Y. Barakat K.M., A.N.S. Physiological Response, Antibacterial Activity and Cinnamaldehyde Production by a Marine Streptomyces Chartreusis. **2016**, *10*.
20. Niu, S.; Li, S.; Chen, Y.; Tian, X.; Zhang, H.; Zhang, G.; Zhang, W.; Yang, X.; Zhang, S.; Ju, J.; et al. Lobophorins e and F, New Spirotetronate Antibiotics from a South China Sea-Derived Streptomyces Sp. SCSIO 01127. *J. Antibiot. (Tokyo).* **2011**, *64*, 711–716, doi:10.1038/ja.2011.78.
21. Rajan, B.M.; Kannabiran, K. Antibiotic Potency of 2,4-Dichloro-5-Sulfamoyl Benzoic Acid Extracted from Marine Bacterium Streptomyces Sp. VITBRK3 against Methicillin Resistant Staphylococcus Aureus. *Der Pharm. Lett.* **2015**, *7*, 244–252.
22. Song, Y.; Yang, J.; Yu, J.; Li, J.; Yuan, J.; Wong, N.-K.; Ju, J. Chlorinated Bis-Indole Alkaloids from Deep-Sea Derived Streptomyces Sp. SCSIO 11791 with Antibacterial and Cytotoxic Activities. *J. Antibiot. (Tokyo).* **2020**, *73*, 542–547.
23. Siddharth, S.; Rai V, R. Isolation and Characterization of Bioactive Compounds with Antibacterial, Antioxidant and Enzyme Inhibitory Activities from Marine-Derived Rare Actinobacteria, Nocardiopsis Sp. SCA21. *Microb. Pathog.* **2019**, *137*, doi:10.1016/j.micpath.2019.103775.
24. León, J.; Aponte, J.J.; Rojas, R.; Cuadra, D.; Ayala, N.; Tomás, G.; Guerrero, M. Study of Marine Actinomycetes Isolated from the Central Coast of Peru and Their Antibacterial Activity against Methicillin-Resistant Staphylococcus Aureus and Vancomycin-Resistant Enterococcus Faecalis [Estudio de Actinomicetos Marinos Aislados de La Cost. *Rev. Peru. Med. Exp. Salud Publica* **2011**, *28*, 237–246.

25. Norouzi, H.; Khorasgani, M.R.; Danesh, A. Anti-MRSA Activity of a Bioactive Compound Produced by a Marine Streptomyces and Its Optimization Using Statistical Experimental Design. *Iran. J. Basic Med. Sci.* **2019**, *22*, 1073–1084.
26. Li, S.; Tian, X.; Niu, S.; Zhang, W.; Chen, Y.; Zhang, H.; Yang, X.; Zhang, W.; Li, W.; Zhang, S.; et al. Pseudonocardians A-C, New Diazaanthraquinone Derivatives from a Deap-Sea Actinomycete Pseudonocardia Sp. SCSIO 01299. *Mar. Drugs* **2011**, *9*, 1428–1439, doi:10.3390/md9081428.
27. Shao, M.; Ma, J.; Li, Q.; Ju, J. Identification of the Anti-Infective Aborycin Biosynthetic Gene Cluster from Deep-Sea-Derived Streptomyces Sp. SCSIO ZS0098 Enables Production in a Heterologous Host. *Mar. Drugs* **2019**, *17*, doi:10.3390/md17020127.
28. Padmanaban, V.P.; Verma, P.; Venkatabaskaran, S.; Keppayan, T.; Gopal, D.; Sekar, A.K.; Ramalingam, K. Antimicrobial Potential and Taxonomic Investigation of Piezotolerant Streptomyces Sp NIOT-Ch-40 Isolated from Deepsea Sediment. *WORLD J. Microbiol. Biotechnol.* **2017**, *33*, doi:10.1007/s11274-016-2193-2.
29. Sunga, M.J.; Teisan, S.; Tsueng, G.; Macherla, V.R.; Lam, K.S. Seawater Requirement for the Production of Lipoxazolidinones by Marine Actinomycete Strain NPS8920. *J. Ind. Microbiol. Biotechnol.* **2008**, *35*, 761–765, doi:10.1007/s10295-008-0344-7.
30. Hu, Y.; Wang, M.; Wu, C.; Tan, Y.; Li, J.; Hao, X.; Duan, Y.; Guan, Y.; Shang, X.; Wang, Y.; et al. Identification and Proposed Relative and Absolute Configurations of Niphimycins C-E from the Marine-Derived Streptomyces Sp IMB7-145 by Genomic Analysis. *J. Nat. Prod.* **2018**, *81*, 178–187, doi:10.1021/acs.jnatprod.7b00859.
31. Song, Y.; Liu, G.; Li, J.; Huang, H.; Zhang, X.; Zhang, H.; Ju, J. Cytotoxic and Antibacterial Angucycline- and Prodigiosin-Analogues from the Deep-Sea Derived Streptomyces Sp. SCSIO 11594. *Mar. Drugs* **2015**, *13*, 1304–1316, doi:10.3390/md13031304.
32. Rajan, B.M.; Kannabiran, K. Extraction and Identification of Antibacterial Secondary Metabolites from Marine Streptomyces Sp. VITBRK2. *Int. J. Mol. Cell. Med.* **2014**, *3*, 130–137.
33. Paderog, M.J. V; Suarez, A.F.L.; Sabido, E.M.; Low, Z.J.; Saludes, J.P.; Dalisay, D.S. Anthracycline Shunt Metabolites From Philippine Marine Sediment-Derived Streptomyces Destroy Cell Membrane Integrity of Multidrug-Resistant Staphylococcus Aureus. *Front. Microbiol.* **2020**, *11*, doi:10.3389/fmicb.2020.00743.
34. Thosar, A.; Satpathy, P.; Jemimah Naine, S.; Devi, C.S. Marine Streptomyces Sp. Vitasp as a Source of New Bioactive Secondary Metabolites. *Curr. Bioact. Compd.* **2020**, *16*, 611–617, doi:10.2174/1573407214666180904100049.
35. Wu, C.; Tan, Y.; Gan, M.; Wang, Y.; Guan, Y.; Hu, X.; Zhou, H.; Shang, X.; You, X.; Yang, Z.; et al. Identification of Elaiophylin Derivatives from the Marine-Derived Actinomycete Streptomyces Sp. 7-145 Using PCR-Based Screening. *J. Nat. Prod.* **2013**, *76*, 2153–2157, doi:10.1021/np4006794.
36. Contreras-Castro, L.; Martínez-García, S.; Cancino-Diaz, J.C.; Maldonado, L.A.; Hernández-Guerrero, C.J.; Martínez-Díaz, S.F.; González-Acosta, B.; Quintana, E.T. Marine Sediment Recovered Salinispora Sp. Inhibits the Growth of Emerging Bacterial Pathogens and Other Multi-Drug-Resistant Bacteria. *Polish J. Microbiol.* **2020**, *69*, 321–330, doi:10.33073/pjm-2020-035.
37. Chung, B.; Kwon, O.-S.; Shin, J.; Oh, K.-B. Antibacterial Activity and Mode of Action of Lactoquinomycin A from Streptomyces Bacillaris. *Mar. Drugs* **2020**, *19*, doi:10.3390/md19010007.
38. Akhter, N.; Liu, Y.; Auckloo, B.N.; Shi, Y.; Wang, K.; Chen, J.; Wu, X.; Wu, B. Stress-Driven Discovery of New Angucycline-Type Antibiotics from a Marine Streptomyces Pratensis NA-ZhouS1. *Mar. Drugs* **2018**, *16*, doi:10.3390/md16090331.

39. Wang, D.; Wang, C.; Gui, P.; Liu, H.; Khalaf, S.M.H.; Elsayed, E.A.; Wadaan, M.A.M.; Hozzein, W.N.; Zhu, W. Identification, Bioactivity, and Productivity of Actinomycins from the Marine-Derived Streptomyces Heliomycini. *Front. Microbiol.* **2017**, *8*, doi:10.3389/fmicb.2017.01147.
40. Benita Mercy R., K.K. Identification of Antibacterial Secondary Metabolite from Marine Streptomyces Sp. VITBRK4 and Its Activity against Drug Resistant Gram Positive Bacteria. **2013**, *5*.
41. Abdulkhair, W.M.; Alghuthaymi, M.A. Double Inhibitory Effect of Extracellular Protein of Marine Streptomyces Tendae against Different Strains of MRSA. *Der Pharm. Lett.* **2016**, *8*, 11–20.
42. Mohseni, M.; Norouzi, H.; Hamedi, J.; Roohi, A. Screening of Antibacterial Producing Actinomycetes from Sediments of the Caspian Sea. *Int. J. Mol. Cell. Med.* **2013**, *2*, 64–71.
43. Shanthi, J.; Senthil, A.; Gopikrishnan, V.; Balagurunathan, R. Characterization of a Potential β-Lactamase Inhibitory Metabolite from a Marine Streptomyces Sp. PM49 Active Against Multidrug-Resistant Pathogens. *Appl. Biochem. Biotechnol.* **2015**, *175*, 3696–3708, doi:10.1007/s12010-015-1538-x.
44. Liang, Y.; Chen, L.; Ye, X.; Anjum, K.; Lian, X.-Y.; Zhang, Z. New Streptophenazines from Marine Streptomyces Sp 182SMLY. *Nat. Prod. Res.* **2017**, *31*, 411–417, doi:10.1080/14786419.2016.1169419.
45. Wang, Q.; Zhang, Y.; Wang, M.; Tan, Y.; Hu, X.; He, H.; Xiao, C.; You, X.; Wang, Y.; Gan, M. Neo-Actinomycins A and B, Natural Actinomycins Bearing the 5H-Oxazolo{[}4,5-b] Phenoxazine Chromophore, from the Marine-Derived Streptomyces Sp IMB094. *Sci. Rep.* **2017**, *7*, doi:10.1038/s41598-017-03769-8.
46. Song, Y.; Huang, H.; Chen, Y.; Ding, J.; Zhang, Y.; Sun, A.; Zhang, W.; Ju, J. Cytotoxic and Antibacterial Marfuraquinocins from the Deep South China Sea-Derived Streptomyces Niveus Scsio 3406. *J. Nat. Prod.* **2013**, *76*, 2263–2268, doi:10.1021/np4006025.
47. Wu, Z.; Li, S.; Li, J.; Chen, Y.; Saurav, K.; Zhang, Q.; Zhang, H.; Zhang, W.; Zhang, W.; Zhang, S.; et al. Antibacterial and Cytotoxic New Napyradiomycins from the Marine-Derived Streptomyces Sp. SCSIO 10428. *Mar. Drugs* **2013**, *11*, 2113–2125, doi:10.3390/md11062113.
48. Hughes, C.C.; Kauffman, C.A.; Jensen, P.R.; Fenical, W. Structures, Reactivities, and Antibiotic Properties of the Marinopyrroles A-F. *J. Org. Chem.* **2010**, *75*, 3240–3250, doi:10.1021/jo1002054.
49. Haste, N.M.; Farnaes, L.; Perera, V.R.; Fenical, W.; Nizet, V.; Hensler, M.E. Bactericidal Kinetics of Marine-Derived Napyradiomycins against Contemporary Methicillin-Resistant Staphylococcus Aureus. *Mar. Drugs* **2011**, *9*, 680–689, doi:10.3390/md9040680.
50. Yuan, X.W.; Yang, R.L.; Cao, X.; Gao, J.J. Taxonomic Identification of a Novel Strain of Streptomyces Cavourensis Subsp. Washingtonensis, ACMA006, Exhibiting Antitumor and Antibacteria Activity. *Drug Discov. & Ther.* **2010**, *4*, 405–411.
51. Cumsille, A.; Undabarrena, A.; Gonzalez, V.; Claverias, F.; Rojas, C.; Camara, B. Biodiversity of Actinobacteria from the South Pacific and the Assessment of Streptomyces Chemical Diversity with Metabolic Profiling. *Mar. Drugs* **2017**, *15*, doi:10.3390/md15090286.
52. Zhang, W.; Liu, Z.; Li, S.; Lu, Y.; Chen, Y.; Zhang, H.; Zhang, G.; Zhu, Y.; Zhang, G.; Zhang, W.; et al. Fluostatins I-K from the South China Sea-Derived Micromonospora Rosaria SCSIO N160. *J. Nat. Prod.* **2012**, *75*, 1937–1943, doi:10.1021/np300505y.
53. Sun, P.; Maloney, K.N.; Nam, S.-J.; Haste, N.M.; Raju, R.; Aalbersberg, W.; Jensen, P.R.; Nizet, V.; Hensler, M.E.; Fenical, W. Fijimycins A-C, Three Antibacterial Etamycin-Class Depsipeptides from a Marine-Derived Streptomyces Sp. *BIOORGANIC \& Med. Chem.* **2011**, *19*, 6557–6562, doi:10.1016/j.bmc.2011.06.053.
54. Wahaab, F.; Subramaniam, K. Bioprospecting Marine Actinomycetes for Multidrug-Resistant Pathogen Control from Rameswaram Coastal Area, Tamil Nadu, India. *Arch. Microbiol.* **2018**,

200, 57–71, doi:10.1007/s00203-017-1417-7.

55. Huang, P.; Xie, F.; Ren, B.; Wang, Q.; Wang, J.; Wang, Q.; Abdel-Mageed, W.M.; Liu, M.; Han, J.; Oyeleye, A.; et al. Anti-MRSA and Anti-TB Metabolites from Marine-Derived Verrucosispora Sp. MS100047. *Appl. Microbiol. Biotechnol.* **2016**, *100*, 7437–7447, doi:10.1007/s00253-016-7406-y.
56. Zhang S. Xie Q., S.C.T.X.-P.G.C.Q.X.Z.H.J.J. Cytotoxic Kendomycins Containing the Carbacylic Ansa Scaffold from the Marine-Derived Verrucosispora Sp. SCSIO 07399. **2019**, *82*.
57. Meena, B.; Anburajan, L.; Vinithkumar, N. V; Kirubagaran, R.; Dharani, G. Biodiversity and Antibacterial Potential of Cultivable Halophilic Actinobacteria from the Deep Sea Sediments of Active Volcanic Barren Island. *Microb. Pathog.* **2019**, *132*, 129–136, doi:10.1016/j.micpath.2019.04.043.
58. Jiang, X.; Zhang, Q.; Zhu, Y.; Nie, F.; Wu, Z.; Yang, C.; Zhang, L.; Tian, X.; Zhang, C. Isolation, Structure Elucidation and Biosynthesis of Benzo[b]Fluorene Nenestatin A from Deep-Sea Derived Micromonospora Echinospora SCSIO 04089. *Tetrahedron* **2017**, *73*, 3585–3590, doi:10.1016/j.tet.2017.03.054.
59. Rajan, B.M.; Kannabiran, K. Antagonistic Activity of Marine Streptomyces Sp. VITBRK1 on Drug Resistant Gram Positive Cocci. *Der Pharm. Lett.* **2013**, *5*, 185–191.
60. Kokare, C.R.; Mahadik, K.R.; Kadam, S.S.; Chopade, B.A. Isolation, Characterization and Antimicrobial Activity of Marine Halophilic Actinopolyspora Species AH1 from the West Coast of India. *Curr. Sci.* **2004**, *86*, 593–597.
61. Ryu, M.-J.; Hillman, P.F.; Lee, J.; Hwang, S.; Lee, E.-Y.; Cha, S.-S.; Yang, I.; Oh, D.-C.; Nam, S.-J.; Fenical, W. Antibacterial Meroterpenoids, Merochlorins G-J from the Marine Bacterium Streptomyces Sp. *Mar. Drugs* **2021**, *19*.
62. Yang, W.Z.; Liang, G.J.; Sun, Y.; Gong, Z.J. Bioactive Secondary Metabolites from Marine Streptomyces Griseorubens F8: Isolation, Identification and Biological Activity Assay. *J. Mar. Sci. Eng.* **2021**, *9*.
63. Sathish Kumar, S.R.; Kokati Venkata Bhaskara, R. In-Vitro Antimicrobial Activity of Marine Actinobacteria against Multidrug Resistance Staphylococcus Aureus. *Asian Pac. J. Trop. Biomed.* **2012**, *2*, S1802–S1807, doi:10.1016/S2221-1691(12)60498-5.
64. Sabido, E.M.; Tenebro, C.P.; Trono, D.J.V.L.; Vicera, C.V.B.; Leonida, S.F.; Maybay, J.J.W.B.; Reyes-Salarda, R.R.; Amago, D.S.; Aguadera, A.M.; Octaviano, M.C.; et al. Insights into the Variation in Bioactivities of Closely Related Streptomyces Strains from Marine Sediments of the Visayan Sea against ESKAPE and Ovarian Cancer. *Mar. Drugs* **2021**, *19*.
65. Tenebro, C.P.; Trono, D.J.V.L.; Vicera, C.V.B.; Sabido, E.M.; Ysulat J, J.A.; Macaspac, A.J.M.; Tampus, K.A.; Fabrigar, T.A.P.; Saludes, J.P.; Dalisay, D.S. Multiple Strain Analysis of Streptomyces Species from Philippine Marine Sediments Reveals Intraspecies Heterogeneity in Antibiotic Activities. *Sci. Rep.* **2021**, *11*, 17544.
66. Mane, M.; Mahadik, K.; Kokare, C. Purification, Characterization and Applications of Thermostable Alkaline Protease from Marine Streptomyces Sp. D1. *Int. J. Pharma Bio Sci.* **2013**, *4*, 572–582.
67. Shin, H.J.; Lee, H.-S.; Lee, D.-S. The Synergistic Antibacterial Activity of 1-Acetyl-Beta-Carboline and Beta-Lactams Against Methicillin-Resistant Staphylococcus Aureus (MRSA). *J. Microbiol. Biotechnol.* **2010**, *20*, 501–505, doi:10.4014/jmb.0910.10019.
68. Ouchene, R.; Intertaglia, L.; Zaatout, N.; Kecha, M.; Suzuki, M.T. Selective Isolation, Antimicrobial Screening and Phylogenetic Diversity of Marine Actinomycetes Derived from the Coast of Bejaia City (Algeria), a Polluted and Microbiologically Unexplored Environment. *J. Appl. Microbiol.* **2022**, *132*, 2870–2882.
69. Kim, M.C.; Li, Z.; Cullum, R.; Molinski, T.F.; Eid, M.A.G.; Hebishy, A.M.S.; Faraag, A.H.I.;

Abdel Moneim, A.E.; Abdelfattah, M.S.; Fenical, W. Chlororesistoflavins A and B, Chlorinated Benzopyrene Antibiotics Produced by the Marine-Derived Actinomycete Streptomyces Sp. Strain EG32. *J. Nat. Prod.* **2022**, *85*, 270–275.

70. Mani, A.; Ravi, L.; Krishnan, K. Antibacterial and Antifungal Potential of Marine Streptomyces Sp. Vitak1 Derived Novel Compound Pyrrolidinyl-Hexadeca-Heptaenone by in Silico Docking Analysis. *Res. J. Pharm. Technol.* **2018**, *11*, 1901–1908, doi:10.5958/0974-360X.2018.00353.0.
71. Zhang D. Yi W., G.H.Z.Z.W.B. Bioactive Streptoglutarimides A-J from the Marine-Derived Streptomyces Sp. ZZ741. *1. NA, A.-D.; GA, E.; AM, G.; MV, A.; V, D.; K, P. Chem. Const. Streptomyces sp. strain Al-Dhabi-97 Isol. from Mar. Reg. Saudi Arab. with Antibact. Anticancer Prop. J. Infect. Public Heal. 2020, 13, 235–243.* **2019**, *82*.
72. Zhou, B.; Huang, Y.; Zhang, H.-J.; Li, J.-Q.; Ding, W. Nitricquinomycins A-C, Uncommon Naphthopyrrolediones from the Streptomyces Sp. ZS-A45. *Tetrahedron* **2019**, *75*, 3958–3961, doi:10.1016/j.tet.2019.05.060.
73. Thi, D.P.; Mai, H.D.T.; Cao, D.D.; Thi, Q.V.; Nguyen, M.A.; Thi, H.M. Le; Tran, D.T.; Chau, V.M.; Pham, V.C. Novel 1,3-Benzodioxole From Marine-Derived Actinomycete in East Vietnam Sea. *Nat. Prod. Commun.* **2020**, *15*, doi:10.1177/1934578X20920042.
74. Zhou, B.; Ji, Y.-Y.; Zhang, H.-J.; Shen, L. Gephyyamycin and Cysrabelomycin, Two New Angucyclinone Derivatives from the Streptomyces Sp. HN-A124. *Nat. Prod. Res.* **2021**, *35*, 2117–2122.
75. Undabarrena, A.; Beltrametti, F.; Claverías, F.P.; González, M.; Moore, E.R.B.; Seeger, M.; Cámara, B. Exploring the Diversity and Antimicrobial Potential of Marine Actinobacteria from the Comau Fjord in Northern Patagonia, Chile. *Front. Microbiol.* **2016**, *7*, 1135, doi:10.3389/fmicb.2016.01135.
76. Manikandan, M.; Gowdaman, V.; Duraimurugan, K.; Prabagaran, S.R. Taxonomic Characterization and Antimicrobial Compound Production from Streptomyces Chumphonensis BDK01 Isolated from Marine Sediment. *3 BIOTECH* **2019**, *9*, doi:10.1007/s13205-019-1687-7.
77. Iniguez-Martinez, A.M.; Cardoso-Martinez, F.; de la Rosa, J.; Cueto, M.; Diaz-Marrero, A.; Darias, J.; Becerril-Espinosa, A.; Plata-Rosas, L.J.; Soria-Mercado, I.E. Compounds Isolated from Salinispora Arenicola of the Gulf of California, Mexico. *Rev. Biol. Mar. Oceanogr.* **2016**, *51*, 161–170, doi:10.4067/S0718-19572016000100015.
78. Dalisay, D.S.; Williams, D.E.; Wang, X.L.; Centko, R.; Chen, J.; Andersen, R.J. Marine Sediment-Derived Streptomyces Bacteria from British Columbia, Canada Are a Promising Microbiota Resource for the Discovery of Antimicrobial Natural Products. *PLoS One* **2013**, *8*, doi:10.1371/journal.pone.0077078.
79. Liang, Y.; Xie, X.; Chen, L.; Yan, S.; Ye, X.; Anjum, K.; Huang, H.; Lian, X.; Zhang, Z. Bioactive Polycyclic Quinones from Marine Streptomyces Sp. 182SMLY. *Mar. Drugs* **2016**, *14*, doi:10.3390/md14010010.
80. Sujatha, P.; Bapi Raju, K.V.V.S.N.; Ramana, T. Studies on a New Marine Streptomycete BT-408 Producing Polyketide Antibiotic SBR-22 Effective against Methicillin Resistant Staphylococcus Aureus. *Microbiol. Res.* **2005**, *160*, 119–126, doi:10.1016/j.micres.2004.10.006.
81. Pan, H.-Q.; Zhang, S.-Y.; Wang, N.; Li, Z.-L.; Hua, H.-M.; Hu, J.-C.; Wang, S.-J. New Spirotetronate Antibiotics, Lobophorins H and I, from a South China Sea-Derived Streptomyces Sp. 12A35. *Mar. Drugs* **2013**, *11*, 3891–3901, doi:10.3390/md11103891.
82. Arasu, M. V; Duraipandiyan, V.; Ignacimuthu, S. Antibacterial and Antifungal Activities of Polyketide Metabolite from Marine Streptomyces Sp. AP-123 and Its Cytotoxic Effect. *Chemosphere* **2013**, *90*, 479–487, doi:10.1016/j.chemosphere.2012.08.006.

83. Song, Y.; Li, Q.; Liu, X.; Chen, Y.; Zhang, Y.; Sun, A.; Zhang, W.; Zhang, J.; Ju, J. Cyclic Hexapeptides from the Deep South China Sea-Derived Streptomyces Scopuliridis SCSIO ZJ46 Active against Pathogenic Gram-Positive Bacteria. *J. Nat. Prod.* **2014**, *77*, 1937–1941, doi:10.1021/np500399v.
84. Attimarad, S.L.; Gaviraj, E.N.; Nagesh, C.; Kugaji, M.S.; Sutar, R.S. Screening, Isolation and Purification of Antibiotic(s) from Marine Actinomycetes. *Int. J. Res. Ayurveda Pharm.* **2012**, *3*, 447–453.
85. Suthindhiran, K.; Kannabiran, K. Diversity and Exploration of Bioactive Marine Actinomycetes in the Bay of Bengal of the Puducherry Coast of India. *Indian J. Microbiol.* **2010**, *50*, 76–82, doi:10.1007/s12088-010-0048-3.
86. Saurav, K.; Kannabiran, K. In Vitro Activity of 5-(2,4-Dimethylbenzyl) Pyrrolidin-2-One Extracted from Marine Streptomyces VITSVK5 Spp. against Fungal and Bacterial Human Pathogens. *Rev. Iberoam. Micol.* **2012**, *29*, 29–33, doi:10.1016/j.riam.2011.06.008.
87. Cho, E.; Kwon, O.-S.; Chung, B.; Lee, J.; Sun, J.; Shin, J.; Oh, K.-B. Antibacterial Activity of Chromomycins from a Marine-Derived Streptomyces Microflavus. *Mar. Drugs* **2020**, *18*, doi:10.3390/md18100522.
88. León, J.; Liza, L.; Soto, I.; Cuadra, D.; Patiño, L.; Zerpa, R. Bioactives Actinomycetes of Marine Sediment from the Central Coast of Peru [Actinomycetes Bioactivos de Sedimento Marino de La Costa Central Del Perú]. *Rev. Peru. Biol.* **2007**, *14*, 259–270.
89. Chen, M.; Chai, W.; Zhu, R.; Song, T.; Zhang, Z.; Lian, X.-Y. Streptopyrazinones A-D, Rare Metabolites from Marine-Derived Streptomyces Sp ZZ446. *Tetrahedron* **2018**, *74*, 2100–2106, doi:10.1016/j.tet.2018.03.028.
90. Yang N., S.F. Bioprospecting of Novel and Bioactive Compounds from Marine Actinomycetes Isolated from South China Sea Sediments. **2018**, *75*.
91. Iniyan, A.M.; Sudarman, E.; Wink, J.; Kannan, R.R.; Vincent, S.G.P. Ala-Geninthiocin, a New Broad Spectrum Thiopeptide Antibiotic, Produced by a Marine Streptomyces Sp. ICN19. *J. Antibiot. (Tokyo).* **2019**, *72*, 99–105, doi:10.1038/s41429-018-0115-2.
92. Kwon, H.C.; Kauffman, C.A.; Jensen, P.R.; Fenical, W. Marinomycins A-D, Antitumor-Antibiotics of a New Structure Class from a Marine Actinomycete of the Recently Discovered Genus "Marinispora". *J. Am. Chem. Soc.* **2006**, *128*, 1622–1632.
93. Jiang, L.; Huang, P.; Ren, B.; Song, Z.; Zhu, G.; He, W.; Zhang, J.; Oyeleye, A.; Dai, H.; Zhang, L.; et al. Antibacterial Polyene-Polyol Macrolides and Cyclic Peptides from the Marine-Derived Streptomyces Sp. MS110128. *Appl. Microbiol. Biotechnol.* **2021**, *105*, 4975–4986.
94. Jagan Mohan, Y.S.Y.V.; Sirisha, B.; Haritha, R.; Ramana, T. Selective Screening, Isolation and Characterization of Antimicrobial Agents from Marine Actinomycetes. *Int. J. Pharm. Pharm. Sci.* **2013**, *5*, 443–449.
95. Setiawati, S.; Nuryastuti, T.; Sholikhah, E.N.; Lisdiyanti, P.; Pratiwi, S.U.T.; Sulistiyani, T.R.; Ratnakomala, S.; Jumina; Mustofa The Potency of Actinomycetes Extracts Isolated from Pramuka Island, Jakarta, Indonesia as Antimicrobial Agents. *Biodiversitas* **2021**, *22*, 1104–1111, doi:10.13057/biodiv/d220304.
96. Miao, L.; Qian, S.; Qi, S.; Jiang, W.; Dong, K. Culture Medium Optimization and Active Compounds Investigation of an Anti-Quorum Sensing Marine Actinobacterium Nocardiopsis Dassonvillei JS106. *Microbiol. (Russian Fed.* **2021**, *90*, 112–123, doi:10.1134/S0026261721010070.
97. Bauermeister, A.; Pereira, F.; Grilo, I.R.; Godinho, C.C.; Paulino, M.; Almeida, V.; Gobbo-Neto, L.; Prieto-Davo, A.; Sobral, R.G.; Lopes, N.P.; et al. Intra-Clade Metabolomic Profiling of MAR4 Streptomyces from the Macaronesia Atlantic Region Reveals a Source of Anti-Biofilm Metabolites. *Environ. Microbiol.* **2019**, *21*, 1099–1112, doi:10.1111/1462-2920.14529.
98. Huang, H.; Song, Y.; Li, X.; Wang, X.; Ling, C.; Qjn, X.; Zhou, Z.; Li, Q.; Wei, X.; Ju, J.

Abyssomicin Monomers and Dimers from the Marine-Derived Streptomyces Koyangensis SCSIO 5802. *J. Nat. Prod.* **2018**, *81*, 1892–1898, doi:10.1021/acs.jnatprod.8b00448.
99. Song Y. Li Q., Q.F.S.C.L.H.W.X.W.N.-K.Y.L.Z.Y.S.M.J.J. Neoabyssomicins A–C, Polycyclic Macrolactones from the Deep-Sea Derived Streptomyces Koyangensis SCSIO 5802. **2017**, *73*.
100. Zhang, Z.; Chen, L.; Zhang, X.; Liang, Y.; Anjum, K.; Chen, L.; Lian, X.-Y. Bioactive Bafilomycins and a New N -Arylpyrazinone Derivative from Marine-Derived Streptomyces SpHZP-2216E. *Planta Med.* **2017**, *83*, 1405–1411, doi:10.1055/s-0043-111897.
101. Djinni I. Defant A., K.M.M.I. Metabolite Profile of Marine-Derived Endophytic Streptomyces Sundarbansensis WR1L1S8 by Liquid Chromatography-Mass Spectrometry and Evaluation of Culture Conditions on Antibacterial Activity and Mycelial Growth. **2014**, *116*.
102. Zhang, X.; Chen, L.; Chai, W.; Lian, X.-Y.; Zhang, Z. A Unique Indolizinium Alkaloid Streptopertusacin A and Bioactive Bafilomycins from Marine-Derived Streptomyces Sp HZP-2216E. *Phytochemistry* **2017**, *144*, 119–126, doi:10.1016/j.phytochem.2017.09.010.
103. Brana, A.F.; Sarmiento-Vizcaino, A.; Perez-Victoria, I.; Martin, J.; Otero, L.; Jose Palacios-Gutierrez, J.; Fernandez, J.; Mohamedi, Y.; Fontanil, T.; Salmon, M.; et al. Desertomycin G, a New Antibiotic with Activity against Mycobacterium Tuberculosis and Human Breast Tumor Cell Lines Produced by Streptomyces Althioticus MSM3, Isolated from the Cantabrian Sea Intertidal Macroalgae Ulva Sp. *Mar. Drugs* **2019**, *17*, doi:10.3390/md17020114.
104. Djinni, I.; Defant, A.; Kecha, M.; Mancini, I. Antibacterial Polyketides from the Marine Alga-Derived Endophitic Streptomyces Sundarbansensis: A Study on Hydroxypyrone Tautomerism. *Mar. Drugs* **2013**, *11*, 124–135, doi:10.3390/md11010124.
105. Katif, C.; Chilczuk, T.; Sabour, B.; Belattmania, Z.; Hilmi, A.; Niedermeyer, T.H.J.; Barakate, M. Isolation and Structure Elucidation of Desferrioxamine B and the New Desferrioxamine B2 Antibiotics from a Brown Marine Macroalga Carpodesmia Tamariscifolia Associated Streptomyces Isolate. *BIOINTERFACE Res. Appl. Chem.* **2022**, *12*, 5647–5662.
106. Rajivgandhi, G.; Vijayan, R.; Kannan, M.; Santhanakrishnan, M.; Manoharan, N. Molecular Characterization and Antibacterial Effect of Endophytic Actinomycetes Nocardiopsis Sp. GRG1 (KT235640) from Brown Algae against MDR Strains of Uropathogens. *Bioact. Mater.* **2016**, *1*, 140–150, doi:10.1016/j.bioactmat.2016.11.002.
107. Eliwa, E.M.; Abdel-Razek, A.S.; Frese, M.; Halawa, A.H.; El-Agrody, A.M.; Bedair, A.H.; Sewald, N.; Shaaban, M. New Naturally Occurring Phenolic Derivatives from Marine Nocardiopsis Sp. AS23C: Structural Elucidation and in Silico Computational Studies. *VIETNAM J. Chem.* **2019**, *57*, 164–174, doi:10.1002/vjch.201900010.
108. Uzair, B.; Menaa, F.; Khan, B.A.; Mohammad, F.V.; Ahmad, V.U.; Djeribi, R.; Menaa, B. Isolation, Purification, Structural Elucidation and Antimicrobial Activities of Kocumarin, a Novel Antibiotic Isolated from Actinobacterium Kocuria Marina CMG S2 Associated with the Brown Seaweed Pelvetia Canaliculata. *Microbiol. Res.* **2018**, *206*, 186–197, doi:10.1016/j.micres.2017.10.007.
109. Sung, A.A.; Gromek, S.M.; Balunas, M.J. Upregulation and Identification of Antibiotic Activity of a Marine-Derived Streptomyces Sp via Co-Cultures with Human Pathogens. *Mar. Drugs* **2017**, *15*, doi:10.3390/md15080250.
110. Govindarajan, G.; Satheeja Santhi, V.; Jebakumar, S.R.D. Antimicrobial Potential of Phylogenetically Unique Actinomycete, Streptomyces Sp. JRG-04 from Marine Origin. *Biologicals* **2014**, *42*, 305–311, doi:10.1016/j.biologicals.2014.08.003.
111. Han, Z.; Xu, Y.; McConnell, O.; Liu, L.; Li, Y.; Qi, S.; Huang, X.; Qian, P. Two Antimycin A Analogues from Marine-Derived Actinomycete Streptomyces Lusitanus. *Mar. Drugs* **2012**, *10*, 668–676, doi:10.3390/md10030668.
112. Qureshi, K.A.; Bholay, A.D.; Rai, P.K.; Mohammed, H.A.; Khan, R.A.; Azam, F.; Jaremko, M.;

Emwas, A.-H.; Stefanowicz, P.; Waliczek, M.; et al. Isolation, Characterization, Anti-MRSA Evaluation, and in-Silico Multi-Target Anti-Microbial Validations of Actinomycin X(2) and Actinomycin D Produced by Novel Streptomyces Smyrnaeus UKAQ_23. *Sci. Rep.* **2021**, *11*, 14539.

113. Tangjitjaroenkun, J.; Pluempanupat, W.; Tangchitcharoenkhul, R.; Yahayo, W.; Supabphol, R. Antibacterial, Antioxidant, Cytotoxic Effects and GC-MS Analysis of Mangrove-Derived Streptomyces Achromogenes TCH4 Extract. *Arch. Biol. Sci.* **2021**, *73*, 223–235.

114. Anggelina A.C. Pringgenies D., S.W.A. Presence of Biosynthetic Gene Clusters (NRPS/PKS) in Actinomycetes of Mangrove Sediment in Semarang and Karimunjawa, Indonesia. **2021**, *19*.

115. Asnani, A.; Purwanti, A.; Bakrudin, W.A.; Anjarwati, D.U. The Production of Streptomyces W-5B Extract for Antibiofilm against Methicillin-Resistant Staphylococcus Aureus. *J. Pure Appl. Microbiol.* **2022**, *16*, 337–346.

116. Braña, A.F.; Sarmiento-Vizcaíno, A.; Pérez-Victoria, I.; Otero, L.; Fernández, J.; Palacios, J.J.; Martín, J.; De La Cruz, M.; Díaz, C.; Vicente, F.; et al. Branimycins B and C, Antibiotics Produced by the Abyssal Actinobacterium Pseudonocardia Carboxydivorans M-227. *J. Nat. Prod.* **2017**, *80*, 569–573, doi:10.1021/acs.jnatprod.6b01107.

117. Ibrahimi, M.; Korichi, W.; Hafidi, M.; Lemee, L.; Ouhdouch, Y.; Loqman, S. Marine Actinobacteria: Screening for Predation Leads to the Discovery of Potential New Drugs against Multidrug-Resistant Bacteria. *ANTIBIOTICS-BASEL* **2020**, *9*, doi:10.3390/antibiotics9020091.

118. Furumai, T.; Eto, K.; Sasaki, T.; Higuchi, H.; Onaka, H.; Saito, N.; Fujita, T.; Naoki, H.; Igarashi, Y. TPU-0037-A, B, C and D, Novel Lydicamycin Congeners with Anti-MRSA Activity from Streptomyces Platensis TP-A0598. *J. Antibiot. (Tokyo).* **2002**, *55*, 873–880.

119. Moghaddam, H.S.; Shahnavaz, B.; Makhdoumi, A.; Iranshahy, M. Evaluating the Effect of Various Bacterial Consortia on Antibacterial Activity of Marine Streptomyces Sp. AC117. *BIOCONTROL Sci. Technol.* **2021**, *31*, 1248–1266.

120. Liu, L.-L.; Xu, Y.; Han, Z.; Li, Y.-X.; Lu, L.; Lai, P.-Y.; Zhong, J.-L.; Guo, X.-R.; Zhang, X.-X.; Qian, P.-Y. Four New Antibacterial Xanthones from the Marine-Derived Actinomycetes Streptomyces Caelestis. *Mar. Drugs* **2012**, *10*, 2571–2583, doi:10.3390/md10112571.

121. Asha Devi, N.K.; Jeyarani, M.; Balakrishnan, K. Isolation and Identification of Marine Actinomycetes and Their Potential in Antimicrobial Activity. *Pakistan J. Biol. Sci.* **2006**, *9*, 470–472, doi:10.3923/pjbs.2006.470.472.

122. Kamarudheen, N.; Naushad, T.; Rao, K.V.B. Biosynthesis, Characterization and Antagonistic Applications of Extracellular Melanin Pigment from Marine Nocardiopsis Sps. *Indian J. Pharm. Educ. Res.* **2019**, *53*, S112–S120, doi:10.5530/ijper.53.2s.55.

123. Nandhagopal, S.; Iniyan, A.M.; Kannan, R.R.; Vincent, S.G.P. In Vivo Evaluation of Anti-MRSA Compound from Streptomyces Collinus ICN1 in Zebrafish Embryos. *INDIAN J. GEO-MARINE Sci.* **2017**, *46*, 1155–1161.

124. Jiao, W.-H.; Yuan, W.; Li, Z.-Y.; Li, J.; Li, L.; Sun, J.-B.; Gui, Y.-H.; Wang, J.; Ye, B.-P.; Lin, H.-W. Anti-MRSA Actinomycins D-1-D-4 from the Marine Sponge-Associated Streptomyces Sp LHW52447. *Tetrahedron* **2018**, *74*, 5914–5919, doi:10.1016/j.tet.2018.08.023.

125. Palomo, S.; González, I.; De la Cruz, M.; Martín, J.; Tormo, J.R.; Anderson, M.; Hill, R.T.; Vicente, F.; Reyes, F.; Genilloud, O. Sponge-Derived Kocuria and Micrococcus Spp. as Sources of the New Thiazolyl Peptide Antibiotic Kocurin. *Mar. Drugs* **2013**, *11*, 1071–1086.

126. Su, P.; Wang, D.-X.; Ding, S.-X.; Zhao, J. Isolation and Diversity of Natural Product Biosynthetic Genes of Cultivable Bacteria Associated with Marine Sponge Mycale Sp. from the Coast of Fujian, China. *Can. J. Microbiol.* **2014**, *60*, 217–225, doi:10.1139/cjm-2013-0785.

127. Cao, D.T.; Tran, V.H.; Vu, V.N.; Mai, H.D.T.; Le, T.H.M.; Vu, T.Q.; Nguyen, H.H.; Chau, V.M.;

Pham, V.C. Antimicrobial Metabolites from a Marine-Derived Actinomycete Streptomyces Sp. G278. *Nat. Prod. Res.* **2019**, *33*, 3223–3230, doi:10.1080/14786419.2018.1468331.
128. Lacret, R.; Oves-Costales, D.; Perez-Victoria, I.; de la Cruz, M.; Diaz, C.; Vicente, F.; Genilloud, O.; Reyes, F. MDN-0171, a New Medermycin Analogue from Streptomyces Albolongus CA-186053. *Nat. Prod. Res.* **2019**, *33*, 66–73, doi:10.1080/14786419.2018.1434636.
129. Zhen X. Gong T., L.F.Z.P.-C.Z.W.-Q.L.Y.Z.P. A New Analogue of Echinomycin and a New Cyclic Dipeptide from a Marine-Derived Streptomyces Sp. LS298. **2015**, *13*.
130. Yu, M.; Li, Y.; Banakar, S.P.; Liu, L.; Shao, C.; Li, Z.; Wang, C. New Metabolites from the Co-Culture of Marine-Derived Actinomycete Streptomyces Rochei MB037 and Fungus Rhinocladiella Similis35. *Front. Microbiol.* **2019**, *10*, doi:10.3389/fmicb.2019.00915.
131. Dong, Y.; Ding, W.; Sun, C.; Ji, X.; Ling, C.; Zhou, Z.; Chen, Z.; Chen, X.; Ju, J. Julichrome Monomers from Marine Gastropod Mollusk-Associated Streptomyces and Stereochemical Revision of Julichromes Q(3.5) and Q(3.3). *Chem. Biodivers.* **2020**, *17*, doi:10.1002/cbdv.202000057.
132. Xu, D.; Nepal, K.K.; Chen, J.; Harmody, D.; Zhu, H.; McCarthy, P.J.; Wright, A.E.; Wang, G. Nocardiopsistins A-C: New Angucyclines with Anti-MRSA Activity Isolated from a Marine Sponge-Derived Nocardiopsis Sp. HB-J378. *Synth. Syst. Biotechnol.* **2018**, *3*, 246–251, doi:10.1016/j.synbio.2018.10.008.
133. Yang, J.; Li, J.; Hu, Y.; Li, L.; Long, L.; Wang, F.; Zhang, S. Characterization of a Thermophilic Hemoglobin-Degrading Protease from Streptomyces Rutgersensis SCSIO 11720 and Its Application in Antibacterial Peptides Production. *Biotechnol. Bioprocess Eng.* **2015**, *20*, 79–90, doi:10.1007/s12257-013-0771-9.
134. Ellis, G.A.; Wyche, T.P.; Fry, C.G.; Braun, D.R.; Bugni, T.S. Solwaric Acids A and B, Antibacterial Aromatic Acids from a Marine Solwaraspora Sp. *Mar. Drugs* **2014**, *12*, 1013–1022, doi:10.3390/md12021013.
135. Miller, B.W.; Torres, J.P.; Tun, J.O.; Flores, M.S.; Forteza, I.; Rosenberg, G.; Haygood, M.G.; Schmidt, E.W.; Concepcion, G.P. Synergistic Anti-Methicillin-Resistant Staphylococcus Aureus (MRSA) Activity and Absolute Stereochemistry of 7,8-Dideoxygriseorhodin C. *J. Antibiot. (Tokyo).* **2020**, *73*, 290–298, doi:10.1038/s41429-019-0275-8.
136. Zhang, Y.; Adnani, N.; Braun, D.R.; Ellis, G.A.; Barns, K.J.; Parker-Nance, S.; Guzei, I.A.; Bugni, T.S. Micromonohalimanes A and B: Antibacterial Halimane-Type Diterpenoids from a Marine Micromonospora Species. *J. Nat. Prod.* **2016**, *79*, 2968–2972, doi:10.1021/acs.jnatprod.6b00555.
137. Asolkar, R.N.; Kirkland, T.N.; Jensen, P.R.; Fenical, W. Arenimycin, an Antibiotic Effective against Rifampin- and Methicillin-Resistant Staphylococcus Aureus from the Marine Actinomycete Salinispora Arenicola. *J. Antibiot. (Tokyo).* **2010**, *63*, 37–39, doi:10.1038/ja.2009.114.
138. Karuppiah, V.; Li, Y.; Sun, W.; Feng, G.; Li, Z. Functional Gene-Based Discovery of Phenazines from the Actinobacteria Associated with Marine Sponges in the South China Sea. *Appl. Microbiol. Biotechnol.* **2015**, *99*, 5939–5950, doi:10.1007/s00253-015-6547-8.
139. Hassan, H.M.; Degen, D.; Jang, K.H.; Ebright, R.H.; Fenical, W. Salinamide F, New Depsipeptide Antibiotic and Inhibitor of Bacterial RNA Polymerase from a Marine-Derived Streptomyces Sp. *J. Antibiot. (Tokyo).* **2015**, *68*, 206–209, doi:10.1038/ja.2014.122.
140. Chen, M.-H.; Lian, Y.-Y.; Fang, D.-S.; Chen, L.; Jia, J.; Zhang, W.-L.; Lin, R.; Xie, Y.; Bi, H.-K.; Jiang, H. Identification and Antimicrobial Properties of a New Alkaloid Produced by Marine-Derived Verrucosispora Sp. FIM06-0036. *Nat. Prod. Res.* **2021**, *35*, 4211–4217.
141. Sandoval-Powers, M.; Králová, S.; Nguyen, G.-S.; Fawwal, D. V; Degnes, K.; Lewin, A.S.; Klinkenberg, G.; Wentzel, A.; Liles, M.R. Streptomyces Poriferorum Sp. Nov., a Novel Marine

Sponge-Derived Actinobacteria Species Expressing Anti-MRSA Activity. *Syst. Appl. Microbiol.* **2021**, *44*, 126244.
142. Hyung-Ju, L.; Joon Soo, A.; Eun Seo, B.; Eunji, C.; Sunghoon, H.; Sang-Jip, N.; Ki-Bong, O.; Sang Kook, L.; Dong-Chan, O. Ligiamycins A and B, Decalin-Amino-Maleimides from the Co-Culture of Streptomyces Sp. and Achromobacter Sp. Isolated from the Marine Wharf Roach, Ligia Exotica. *Mar. Drugs* **2022**, *20*.
143. Santos J.D. Vitorino I., D.L.C.M.D.C.C.B.A.F.P.-M.G.M.I.G.T.J.R.M.J.M.U.R.V.F.M.L.O.M. Bioactivities and Extract Dereplication of Actinomycetales Isolated from Marine Sponges. **2019**, *10*.
144. Gavriilidou, A.; Mackenzie, T.A.; Sánchez, P.; Tormo, J.R.; Ingham, C.; Smidt, H.; Sipkema, D. Bioactivity Screening and Gene-Trait Matching across Marine Sponge-Associated Bacteria. *Mar. Drugs* **2021**, *19*, doi:10.3390/md19020075.
145. Carretero-Molina, D.; Ortiz-López, F.J.; Martín, J.; Oves-Costales, D.; Díaz, C.; de la Cruz, M.; Cautain, B.; Vicente, F.; Genilloud, O.; Reyes, F.; et al. New Napyradiomycin Analogues from Streptomyces Sp. Strain CA-271078. *Mar. Drugs* **2020**, *18*, doi:10.3390/md18010022.
146. Rajasabapathy, R.; Ghadi, S.C.; Manikandan, B.; Mohandass, C.; Surendran, A.; Dastager, S.G.; Meena, R.M.; James, R.A. Antimicrobial Profiling of Coral Reef and Sponge Associated Bacteria from Southeast Coast of India. *Microb. Pathog.* **2020**, *141*, doi:10.1016/j.micpath.2020.103972.
147. Chen, M.-H.; Zhang, W.-L.; Chen, L.; Lin, R.; Xie, Y.; Fang, D.-S.; Lian, Y.-Y.; Jiang, H. Isolation, Purification and Identification of Two New Alkaloids Metabolites from Marine-Derived Verrucosispora Sp. FIM06025. *Nat. Prod. Res.* **2019**, *33*, 2897–2903, doi:10.1080/14786419.2018.1509333.
148. Zhang, X.; Ye, X.; Chai, W.; Lian, X.-Y.; Zhang, Z. New Metabolites and Bioactive Actinomycins from Marine-Derived Streptomyces Sp. ZZ338. *Mar. Drugs* **2016**, *14*, doi:10.3390/md14100181.
149. Cao, D.D.; Do, T.Q.; Mai, H.D.T.; Thi, Q.V.; Nguyen, M.A.; Thi, H.M. Le; Tran, D.T.; Chau, V.M.; Thung, D.C.; Pham, V.C. Antimicrobial Lavandulylated Flavonoids from a Sponge-Derived Actinomycete. *Nat. Prod. Res.* **2020**, *34*, 413–420, doi:10.1080/14786419.2018.1538219.
150. Singh, S.; Prasad, P.; Subramani, R.; Aalbersberg, W. Production and Purification of a Bioactive Substance against Multi-Drug Resistant Human Pathogens from the Marine-Sponge-Derived Salinispora Sp. *Asian Pac. J. Trop. Biomed.* **2014**, *4*, 825–831, doi:10.12980/APJTB.4.2014C1154.
151. Cong Z. Huang X., L.Y.L.Y.W.P.L.S.Y.B.Z.X.H.D.W.J. Cytotoxic Anthracycline and Antibacterial Tirandamycin Analogues from a Marine-Derived Streptomyces Sp. SCSIO 41399. **2019**, *72*.
152. Eltamany, E.E.; Abdelmohsen, U.R.; Ibrahim, A.K.; Hassanean, H.A.; Hentschel, U.; Ahmed, S.A. New Antibacterial Xanthone from the Marine Sponge-Derived Micrococcus Sp. EG45. *Bioorganic Med. Chem. Lett.* **2014**, *24*, 4939–4942, doi:10.1016/j.bmcl.2014.09.040.
153. Kunz, A.L.; Labes, A.; Wiese, J.; Bruhn, T.; Bringmann, G.; Imhoff, J.F. Nature's Lab for Derivatization: New and Revised Structures of a Variety of Streptophenazines Produced by a Sponge-Derived Streptomyces Strain. *Mar. Drugs* **2014**, *12*, 1699–1714, doi:10.3390/md12041699.
154. Abdelmohsen U.R. Pimentel-Elardo S.M., H.A.R.M.A.-E.-E.S.H.A.S.H.U. Isolation, Phylogenetic Analysis and Anti-Infective Activity Screening of Marine Sponge-Associated Actinomycetes. **2010**, *8*.
155. Dharmaraj S., S.A. Bioactive Potential of Streptomyces Associated with Marine Sponges. **2009**, *25*.

156. Elsayed, Y.; Refaat, J.; Abdelmohsen, U.R.; Othman, E.M.; Stopper, H.; Fouad, M.A. Metabolomic Profiling and Biological Investigation of the Marine Sponge-Derived Bacterium Rhodococcus Sp. UA13. *Phytochem. Anal.* **2018**, *29*, 543–548, doi:10.1002/pca.2765.
157. Lacret, R.; Pérez-Victoria, I.; Oves-Costales, D.; de la Cruz, M.; Domingo, E.; Martín, J.; Díaz, C.; Vicente, F.; Genilloud, O.; Reyes, F. MDN-0170, a New Napyradiomycin from Streptomyces Sp. Strain CA-271078. *Mar. Drugs* **2016**, *14*, doi:10.3390/md14100188.
158. Xu, D.; Han, L.; Li, C.; Cao, Q.; Zhu, D.; Barrett, N.H.; Harmody, D.; Chen, J.; Zhu, H.; McCarthy, P.J.; et al. Bioprospecting Deep-Sea Actinobacteria for Novel Anti-Infective Natural Products. *Front. Microbiol.* **2018**, *9*, doi:10.3389/fmicb.2018.00787.
159. Shaala, L.A.; Youssef, D.T.A.; Alzughaibi, T.A.; Elhady, S.S. Antimicrobial Chlorinated 3-Phenylpropanoic Acid Derivatives from the Red Sea Marine Actinomycete Streptomyces Coelicolor LY001. *Mar. Drugs* **2020**, *18*, doi:10.3390/md18090450.
160. Zhang Z. Sibero M.T., K.A.F.K.U.D.I.Y. TMKS8A, an Antibacterial and Cytotoxic Chlorinated α-Lapachone, from a Sea Slug-Derived Actinomycete of the Genus Streptomyces. **2021**, *74*.
161. Newaz A.W. Yong K., L.X.-Y.Z.Z. Streptoindoles A–D, Novel Antimicrobial Indole Alkaloids from the Marine-Associated Actinomycete Streptomyces Sp. ZZ1118. **2021**.
162. Kamarudheen, N.; Rao, K.V.B. Fatty Acyl Compounds from Marine Streptomyces Griseoincarnatus Strain HK12 against Two Major Bio-Film Forming Nosocomial Pathogens; an in Vitro and in Silico Approach. *Microb. Pathog.* **2019**, *127*, 121–130, doi:10.1016/j.micpath.2018.11.050.
163. Hifnawy, M.S.; Hassan, H.M.; Mohammed, R.; Fouda, M.M.; Sayed, A.M.; Hamed, A.A.; AbouZid, S.F.; Rateb, M.E.; Alhadrami, H.A.; Abdelmohsen, U.R. Induction of Antibacterial Metabolites by Co-Cultivation of Two Red-Sea-Sponge-Associated Actinomycetes Micromonospora Sp. UR56 and Actinokinespora Sp. EG49. *Mar. Drugs* **2020**, *18*, doi:10.3390/md18050243.
164. Iniyan, A.M.; Mary, T.R.J.; Joseph, F.-J.R.S.; Kannan, R.R.; Vincent, S.G.P. Cell Wall Distracting Anti-Methicillin-Resistant Staphylococcus Aureus Compound PVI331 from a Marine Sponge Associated Streptomyces. *J. Appl. Biomed.* **2016**, *14*, 273–283, doi:10.1016/j.jab.2016.04.003.
165. Mary, T.R.J.; Kannan, R.R.; Iniyan, A.M.; Ramachandran, D.; Vincent, S.G.P. Cell Wall Distraction and Biofilm Inhibition of Marine Streptomyces Derived Angucycline in Methicillin Resistant Staphylococcus Aureus. *Microb. Pathog.* **2021**, *150*, doi:10.1016/j.micpath.2020.104712.
166. Balasubramanian, S.; Skaf, J.; Holzgrabe, U.; Bharti, R.; Foerstner, K.U.; Ziebuhr, W.; Humeida, U.H.; Abdelmohsen, U.R.; Oelschlaeger, T.A. A New Bioactive Compound From the Marine Sponge-Derived Streptomyces Sp SBT348 Inhibits Staphylococcal Growth and Biofilm Formation. *Front. Microbiol.* **2018**, *9*, doi:10.3389/fmicb.2018.01473.
167. Joseph, F.J.R.S.; Iniyan, A.M.; Vincent, S.G.P. HR-LC-MS Based Analysis of Two Antibacterial Metabolites from a Marine Sponge Symbiont Streptomyces Pharmamarensis ICN40. *Microb. Pathog.* **2017**, *111*, 450–457, doi:10.1016/j.micpath.2017.09.033.
168. Ravikumar S. Gnanadesigan M., S.A.M.N.B.V.M.S. Antagonistic Properties of Seagrass Associated Streptomyces Sp. RAUACT-1: A Source for Anthraquinone Rich Compound. **2012**, *5*.
169. Cristianawati, O.; Sibero, M.T.; Ayuningrum, D.; Nuryadi, H.; Syafitri, E.; Radjasa, O.K.; Riniarsih, I. Screening of Antibacterial Activity of Seagrass-Associated Bacteria from the North Java Sea, Indonesia against Multidrug-Resistant Bacteria. *AACL Bioflux* **2019**, *12*, 1054–1064.
170. Kurnianto M.A. Kusumaningrum H.D., L.H.N.C.E. Antibacterial and Antioxidant Potential of Ethyl Acetate Extract from Streptomyces AIA12 and AIA17 Isolated from Gut of Chanos

Chanos. **2021**, *22*.

171. Vignesh, A.; Ayswarya, S.; Gopikrishnan, V.; Radhakrishnan, M. Bioactive Potential of Actinobacteria Isolated from the Gut of Marine Fishes. *Indian J. Geo-Marine Sci.* **2019**, *48*, 1280–1285.
172. Al-Dhabi, N.A.; Ghilan, A.-K.M.; Esmail, G.A.; Arasu, M.V.; Duraipandiyan, V.; Ponmurugan, K. Bioactivity Assessment of the Saudi Arabian Marine Streptomyces Sp. Al-Dhabi-90, Metabolic Profiling and Its in Vitro Inhibitory Property against Multidrug Resistant and Extended-Spectrum Beta-Lactamase Clinical Bacterial Pathogens. *J. Infect. Public Health* **2019**, *12*, 549–556, doi:10.1016/j.jiph.2019.01.065.
173. Jiang, Y.-J.; Zhang, D.-S.; Zhang, H.-J.; Li, J.-Q.; Ding, W.-J.; Xu, C.-D.; Ma, Z.-J. Medermycin-Type Naphthoquinones from the Marine-Derived Streptomyces Sp XMA39. *J. Nat. Prod.* **2018**, *81*, 2120–2124, doi:10.1021/acs.jnatprod.8b00544.
174. Ding, W.; Dong, Y.; Ju, J.; Li, Q. The Roles of Genes Associated with Regulation, Transportation, and Macrocyclization in Desotamide Biosynthesis in Streptomyces Scopuliridis SCSIO ZJ46. *Appl. Microbiol. Biotechnol.* **2020**, *104*, 2603–2610, doi:10.1007/s00253-020-10414-4.
175. Luo, M.; Tang, L.; Dong, Y.; Huang, H.; Deng, Z.; Sun, Y. Antibacterial Natural Products Lobophorin L and M from the Marine-Derived Streptomyces Sp. 4506. *Nat. Prod. Res.* **2020**, doi:10.1080/14786419.2020.1797730.
176. Haste, N.M.; Perera, V.R.; Maloney, K.N.; Tran, D.N.; Jensen, P.; Fenical, W.; Nizet, V.; Hensler, M.E. Activity of the Streptogramin Antibiotic Etamycin against Methicillin-Resistant Staphylococcus Aureus. *J. Antibiot. (Tokyo).* **2010**, *63*, 219–224, doi:10.1038/ja.2010.22.
177. Yin, Q.; Liang, J.; Zhang, W.; Zhang, L.; Hu, Z.-L.; Zhang, Y.; Xu, Y. Butenolide, a Marine-Derived Broad-Spectrum Antibiofilm Agent Against Both Gram-Positive and Gram-Negative Pathogenic Bacteria. *Mar. Biotechnol.* **2019**, *21*, 88–98, doi:10.1007/s10126-018-9861-1.
