# Supplementary material for: Marine Actinobacteria a New Source of Antibacterial Metabolites to Treat Acne Vulgaris Disease—A Systematic Literature Review
Source: Antibiotics (Basel). 2022 Jul 18;11(7):965. doi: 10.3390/antibiotics11070965 (PMC9311749; doi:10.3390/antibiotics11070965)
Supplement: Supplementary file 1 [file antibiotics-11-00965-s001.zip › Table S3.pdf]

Table S3. Antibacterial activity of actinobacterial crude extracts or compounds presented through inhibition zone (mm).

Genus Name	Pathogen bacteria target	Compounds/extracts	Inhibiton Zone (mm)	Ref.
Streptomyces sp.	Staphylococcus sp.	Long chain fatty acids.	16	[1]
Streptomyces sp.	*S. aureus*	SBSK-67 Fatty acid esters	14	[2]
		SBSK-120 Fatty acid esters	16	
Streptomyces sp.	*S. aureus* ATCC 6538	n-butanol extract 8	14	[3]
		n-butanol extract 9	20	
Streptomyces sp.	*S. aureus*	Aromatic polyketide Arabomycin	17	[4]
Streptomyces sp.	*S. aureus* ATCC 6538	Isolated S8	9	[5]
Streptomyces sp.	*S. aureus*	EtOAc Extract EH1	17	[6]
		EtOAc Extract EH1	14	
Streptomyces sp.	MRSA[1] VRE[3]	Culture supernatant	17	[7]
	S. aureus (ATCC 700699)	Culture supernatant	12	
	S. aureus ATCC 25923	Culture supernatant	21	
Streptomyces sp.	*S. aureus*	EtOAc Extract crude	25	[8]
Streptomyces sp.	*S. aureus*	EMM111<br>EMM112<br>EMM183	17.5<br>35.4<br>35	[9]
	MRSA[1]	EMM111<br>EMM112<br>EMM183	16.3<br>34.6<br>23.26	
Actinomadura sp.	*S. aureus* NBRC12732	Fraction	12.7	[10]

Nocardiopsis sp.	*S. aureus*	Extract SJP1	9	[11]
		Extract SJP4	9	
Streptomyces sp.	MRSE[2] RP62A	Compound (SKC3)	31.25	[12]
Streptomyces sp.	MRSA[1]	PVI401<br>PVI402	0.5<br>2	[13]
Streptomyces sp.	MRSA[1]	EtOAc Extract	19	[14]
Streptomyces sp.	*S. aureus*	PA720[4]	18	[15]
Streptomyces sp.	*S. aureus*	FBA6[5]	11	[16]
Streptomyces sp.	*S. aureus*	RAM25C4[6]	34	[17]
	MRSA[1]		31	
Streptomyces sp.	MRSA[1]		24	[18]
	S. aureus (ATCC 29213)		21	
	S. aureus (ATCC25923)		21	
	S. aureus (ATCC 700699)		13	
Streptomyces sp.	MRSA[1]	Butanolic extract	20	[19]
Unidentified	*S. aureus*	Extract	20	[20]

[1]MRSA: Methicillin resistant *Staphylococcus aureus*. [2]MRSE: Methicillin resistant *Staphylococcus epidermidis*. [3]VRE: vancomycin resistant Enterococci. [4]Hemoglobin degrading protease. [5]Fermentation broth of ACMA006 strain. [6]Isolate RAM25C4.

References

1. Dholakiya, R.N.; Kumar, R.; Mishra, A.; Mody, K.H.; Jha, B. Antibacterial and Antioxidant Activities of Novel Actinobacteria Strain Isolated from Gulf of Khambhat, Gujarat. *Front. Microbiol.* **2017**, *8*, doi:10.3389/fmicb.2017.02420.

2. Ballav, S.; Kerkar, S.; Thomas, S.; Augustine, N. Halophilic and Halotolerant Actinomycetes from a Marine Saltern of Goa, India Producing Anti-Bacterial Metabolites. *J. Biosci. Bioeng.* **2015**, *119*, 323–330, doi:10.1016/j.jbiosc.2014.08.017.

3. Abd-Elnaby, H.; Abo-Elala, G.; Abdel-Raouf, U.; Abd-Elwahab, A.; Hamed, M. Antibacterial and Anticancer Activity of Marine Streptomyces Parvus: Optimization and Application. *Biotechnol. Biotechnol. Equip.* **2016**, *30*, 180–191, doi:10.1080/13102818.2015.1086280.

4. Ahmad, S.; Nazir, M.; Tousif, M.I.; Saleem, M.; Mustafa, R.; Khatoon, T. A New Polyketide Antibiotic from the Marine Bacterium Streptomyces Sp. PGC 32. *Chem. Nat. Compd.* **2019**, *55*, 1–4, doi:10.1007/s10600-019-02602-0.

5. Antoniraj A. Anandan V., G.T.G.A. Isolation of Marine Actinomycetes Associated with the Carangid Fish Alepes Melanoptera, (Swainson, 1839) and an Evaluation of Their Antimicrobial Activity. **2018**, *20*.

6. El-Naggar M.Y. Barakat K.M., A.N.S. Physiological Response, Antibacterial Activity and Cinnamaldehyde Production by a Marine Streptomyces Chartreusis. **2016**, *10*.

7. BM, R.; K, K. Extraction and Identification of Antibacterial Secondary Metabolites from Marine Streptomyces Sp. VITBRK2. *Int. J. Mol. Cell. Med.* **2014**, *3*, 130–137.

8. Thosar, A.; Satpathy, P.; Jemimah Naine, S.; Devi, C.S. Marine Streptomyces Sp. Vitasp as a Source of New Bioactive Secondary Metabolites. *Curr. Bioact. Compd.* **2020**, *16*, 611–617, doi:10.2174/1573407214666180904100049.

9. Ibrahimi, M.; Korichi, W.; Hafidi, M.; Lemee, L.; Ouhdouch, Y.; Loqman, S. Marine Actinobacteria: Screening for Predation Leads to the Discovery of Potential New Drugs against Multidrug-Resistant Bacteria. *Antibiotics* **2020**, *9*, 1–14, doi:10.3390/antibiotics9020091.

10. Kurata, A.; Sugiura, M.; Kokoda, K.; Tsujimoto, H.; Numata, T.; Kato, C.; Nakasone, K.; Kishimoto, N. Taxonomy of Actinomycetes in the Deep-Sea Calyptogena Communities and Characterization of the Antibacterial Compound Produced by Actinomadura Sp DS-MS-114. *Biotechnol. Biotechnol. Equip.* **2017**, *31*, 1000–1006, doi:10.1080/13102818.2017.1342563.

11. Priyanka, S.; Jayashree, M.; Shivani, R.; Anwesha, S.; Bhaskara Rao, K. V.; I, A.E. Characterisation and Identification of Antibacterial Compound from Marine Actinobacteria: In Vitro and in Silico Analysis. *J. Infect. Public Health* **2019**, *12*, 83–89, doi:10.1016/j.jiph.2018.09.005.

12. Balasubramanian, S.; Skaf, J.; Holzgrabe, U.; Bharti, R.; Foerstner, K.U.; Ziebuhr, W.; Humeida, U.H.; Abdelmohsen, U.R.; Oelschlaeger, T.A. A New Bioactive Compound From the Marine Sponge-Derived Streptomyces Sp SBT348 Inhibits Staphylococcal Growth and Biofilm Formation. *Front. Microbiol.* **2018**, *9*, doi:10.3389/fmicb.2018.01473.

13. Joseph, F.J.R.S.; Iniyan, A.M.; Vincent, S.G.P. HR-LC-MS Based Analysis of Two Antibacterial Metabolites from a Marine Sponge Symbiont Streptomyces Pharmamarensis ICN40. *Microb.*

Pathog. **2017**, *111*, 450–457, doi:10.1016/j.micpath.2017.09.033.

14. Benita Mercy, R.; Kannabiran, K. Identification of Antibacterial Secondary Metabolite from Marine Streptomyces Sp. VITBRK4 and Its Activity against Drug Resistant Gram Positive Bacteria. *Int. J. Drug Dev. Res.* **2013**, *5*, 224–232.

15. Yang, J.; Li, J.; Hu, Y.; Li, L.; Long, L.; Wang, F.; Zhang, S. Characterization of a Thermophilic Hemoglobin-Degrading Protease from Streptomyces Rutgersensis SCSIO 11720 and Its Application in Antibacterial Peptides Production. *Biotechnol. Bioprocess Eng.* **2015**, *20*, 79–90, doi:10.1007/s12257-013-0771-9.

16. Yuan, X.W.; Yang, R.L.; Cao, X.; Gao, J.J. Taxonomic Identification of a Novel Strain of Streptomyces Cavourensis Subsp. Washingtonensis, ACMA006, Exhibiting Antitumor and Antibacteria Activity. *Drug Discov. & Ther.* **2010**, *4*, 405–411.

17. Wahaab, F.; Subramaniam, K. Bioprospecting Marine Actinomycetes for Multidrug-Resistant Pathogen Control from Rameswaram Coastal Area, Tamil Nadu, India. *Arch. Microbiol.* **2018**, *200*, 57–71, doi:10.1007/s00203-017-1417-7.

18. Rajan, B.M.; Kannabiran, K. Antagonistic Activity of Marine Streptomyces Sp. VITBRK1 on Drug Resistant Gram Positive Cocci. *Der Pharm. Lett.* **2013**, *5*, 185–191.

19. Katif, C.; Chilczuk, T.; Sabour, B.; Belattmania, Z.; Hilmi, A.; Niedermeyer, T.H.J.; Barakate, M. Isolation and Structure Elucidation of Desferrioxamine B and the New Desferrioxamine B2 Antibiotics from a Brown Marine Macroalga Carpodesmia Tamariscifolia Associated Streptomyces Isolate. *BIOINTERFACE Res. Appl. Chem.* **2022**, *12*, 5647–5662.

20. M, M.; H, N.; J, H.; A, R. Screening of Antibacterial Producing Actinomycetes from Sediments of the Caspian Sea. *Int. J. Mol. Cell. Med.* **2013**, *2*, 64–71.
