# Supplementary material for: Marine Actinobacteria a New Source of Antibacterial Metabolites to Treat Acne Vulgaris Disease—A Systematic Literature Review
Source: Antibiotics (Basel). 2022 Jul 18;11(7):965. doi: 10.3390/antibiotics11070965 (PMC9311749; doi:10.3390/antibiotics11070965)
Supplement: Supplementary file 1 [file antibiotics-11-00965-s001.zip › Table S4.pdf]

Table S4. Antimicrobial activity of actinobacterial crude extracts or compounds presented through inhibition zone (mm).

Genus Name	Pathogen bacteria target	Compounds/extracts	Inhibition zone (-mm)	Ref.
Streptomyces sp.	*S. aureus*	Extracts	17	[1]
Streptomyces sp.	*S. aureus* MTCC 3160	PM1	24	[2]
		PM2	21.6	
		PM3	14.3	
Streptomyces sp.	*S. aureus* 8325	Methanolic extract	17	[3]
Streptomyces sp.	*S. aureus*	EtOAc extract ACT-A2	18	[4]
Streptomyces sp.	*S. aureus*	EtOAc extract	27.3	[5]
Actinopolyspora sp.	*S. aureus*	EtOAc extract	26	
Streptomyces sp.	*S. aureus*	EtOAc extract	14	[6]
Streptomyces sp.	*S. aureus* 457	M10-77	36.75	[7]
	S. aureus	EtOAc extract J378-la	25	[8]
	MRSA[1]	EtOAc extract J378-la	16	
	S. aureus	EtOAc extract N217	14	
	MRSA[1]	EtOAc extract N217	20	

[1] Methicillin resistant *Staphylococcus aureus*.

References

1. Eliwa, E.M.; Abdel-Razek, A.S.; Frese, M.; Halawa, A.H.; El-Agrody, A.M.; Bedair, A.H.; Sewald, N.; Shaaban, M. New Naturally Occurring Phenolic Derivatives from Marine Nocardiopsis Sp. AS23C: Structural Elucidation and in Silico Computational Studies. *VIETNAM J. Chem.* **2019**, *57*, 164–174, doi:10.1002/vjch.201900010.

2. Manikandan, M.; Gowdaman, V.; Duraimurugan, K.; Prabagaran, S.R. Taxonomic

Characterization and Antimicrobial Compound Production from Streptomyces Chumphonensis BDK01 Isolated from Marine Sediment. *3 BIOTECH* **2019**, *9*, doi:10.1007/s13205-019-1687-7.

3. Abdelmohsen, U.R.; Pimentel-Elardo, S.M.; Hanora, A.; Radwan, M.; Abou-El-Ela, S.H.; Ahmed, S.; Hentschel, U. Isolation, Phylogenetic Analysis and Anti-Infective Activity Screening of Marine Sponge-Associated Actinomycetes. *Mar. Drugs* **2010**, *8*, 399–412, doi:10.3390/md8030399.

4. Attimarad S.L. Gaviraj E.N., N.C.K.M.S.S.R.S. Screening, Isolation and Purification of Antibiotic(s) from Marine Actinomycetes. **2012**, *3*.

5. Suthindhiran, K.; Kannabiran, K. Diversity and Exploration of Bioactive Marine Actinomycetes in the Bay of Bengal of the Puducherry Coast of India. *Indian J. Microbiol.* **2010**, *50*, 76–82, doi:10.1007/s12088-010-0048-3.

6. Dharmaraj S., S.A. Bioactive Potential of Streptomyces Associated with Marine Sponges. **2009**, *25*.

7. León, J.; Liza, L.; Soto, I.; Cuadra, D.; Patiño, L.; Zerpa, R. Bioactives Actinomycetes of Marine Sediment from the Central Coast of Peru [Actinomycetes Bioactivos de Sedimento Marino de La Costa Central Del Perú]. *Rev. Peru. Biol.* **2007**, *14*, 259–270.

8. Xu, D.; Han, L.; Li, C.; Cao, Q.; Zhu, D.; Barrett, N.H.; Harmody, D.; Chen, J.; Zhu, H.; McCarthy, P.J.; et al. Bioprospecting Deep-Sea Actinobacteria for Novel Anti-Infective Natural Products. *Front. Microbiol.* **2018**, *9*, doi:10.3389/fmicb.2018.00787.
